# Preparing a Dual-Species *In Vitro* Biofilm Model for Testing Antibiofilm Efficacy

**DOI:** 10.1021/acs.molpharmaceut.5c00798

**Published:** 2025-10-19

**Authors:** Kelli Randmäe, Kairi Lorenz, Marta Putrinš, Tanel Tenson, Karin Kogermann

**Affiliations:** † Institute of Pharmacy, 37546 University of Tartu, Nooruse 1, Tartu 50411, Estonia; ‡ Institute of Technology, 124633 University of Tartu, Nooruse 1, Tartu 50411, Estonia

**Keywords:** in vitro biofilm model, dual-species biofilm, antibiofilm, antibacterial, electrospinning, wound dressings, skin wound infection

## Abstract

All wounds are contaminated, and there is a risk of developing an infection. Furthermore, most wounds contain biofilm and are contaminated by two bacteria, termed dual-species, or more bacteria, termed polybacterial biofilms. New antibacterial and antibiofilm wound care products are constantly being developed to combat this problem. There is a need to develop more biorelevant and reproducible models to test the efficacy of these wound care products. We used an electrospun (ES) gelatin-glucose matrix (Gel-Gluc) as an artificial skin substrate for dual-species biofilm formation using wound pathogens *Staphylococcus aureus*, *Escherichia coli*, and *Pseudomonas aeruginosa*, combining them in pairs. When analyzing the biofilms, selective agars were used to differentiate various bacteria from one another while counting. The developed method supported the growth of dual-species biofilm that contained both bacteria up to 10^8^ CFU/Gel-Gluc after 24 h. Over 48 h, there was a decrease in the number of *S. aureus* in the biofilms. Confocal microscopy imaging allowed monitoring of the location of bacteria in the Gel-Gluc and proved that different species were located closely together. ES polycaprolactone (PCL) fibrous wound dressings containing chloramphenicol (CAM) or ciprofloxacin (CIP), or their pristine analogs, were used to test the model. Both ES fibrous wound dressings were effective in preventing dual-species biofilm formation. PCL-CIP fibrous dressing was also effective in treating biofilms. The efficacy of treatment of *E. coli* varied in different dual-species combinations of *E. coli*. The developed dual-species biofilm model on artificial skin (Gel-Gluc) supported the successful growth of different bacterial combinations and proved to be suitable for testing the efficacy of ES fibrous wound dressings in preventing and treating biofilms.

## Graphical abstract

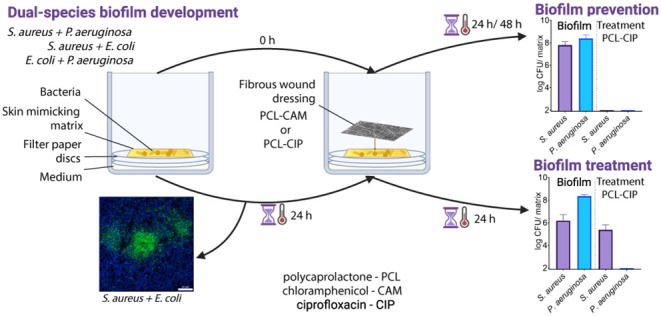

## 1 Introduction

All wounds are contaminated with bacteria, and an infection may develop. Infectious wounds predominantly contain bacteria in the form of biofilms. It has been estimated that approximately 80% of chronic wounds contain biofilms.^1^ These biofilms are frequently polybacterial. For instance, the presence of more than one bacterial species was identified in all venous leg ulcer samples collected from 46 patients.^2^ In another study, it was shown that diabetic foot ulcers contained an average of three bacterial species, with some samples harboring up to eight species.^3^

*Staphylococcus aureus* and *Pseudomonas aeruginosa* are among the most frequently identified bacteria in chronic wound infections. Other bacterial species isolated include Enterococcus sp., Streptococcus sp., and *Escherichia coli*.^4–6^ Therefore, it is often necessary to find a suitable treatment against several pathogens simultaneously. Inhibiting and treating biofilms is challenging^7^ due to the presence of a bacterial self-produced protective extracellular matrix, but in the case of polybacterial infection, the treatment is particularly challenging due to the interactions between bacterial species, which can enhance resistance and tolerance to antimicrobial therapies.^4,8^ For example, it has been shown that polybacterial biofilms significantly impair wound healing compared to their single-species biofilm counterparts, while simultaneously triggering a greater host inflammatory response.^9^

Given the significant role of polybacterial biofilms in chronic wounds, it is crucial to gain a deeper understanding of these wound biofilms and develop strategies to prevent their formation or treat biofilm-related infections. In addition to addressing new fundamental questions about polybacterial biofilms, there is a need to test novel antimicrobial products under these challenging conditions. There is a growing need for effective antimicrobial and antibiofilm wound care products for local use.^10,11^ It is most cost-effective to test the efficacy of these products at the beginning of the development phase under biorelevant conditions. Therefore, there is a need for biorelevant wound infection or biofilm models that are suitable for this type of testing.

Different *in vitro* dual-species and polybacterial (multispecies) biofilm models have been developed previously.^12–17^ These polybacterial models enable us to better understand the interactions between species and compare different treatments when fighting against multiple pathogens at the same time. Although different bacteria grow together in biofilms in nature, it is much more complicated to grow dual-species and polybacterial biofilms in the laboratory. *In vitro* polybacterial biofilm models often suffer from the fact that some bacteria tend to overgrow others, and actual inhibition and treatment effects can only be tested against this single bacterial species.^15,18^ In some cases, biorelevant polybacterial biofilms have been successfully grown,^16^ but the experiments are more expensive to conduct, the models are more complex, and they are not always well-suited for testing various topical wound care products. For example, Lubbock’s biofilm model has enabled the growth of substantially stable polybacterial biofilms using up to four species of bacteria.^14,19^ However, its use for the routine testing of topical formulations requires modifications to improve reproducibility and ease of comparison. Understanding the antibiofilm efficacy of wound products in *in vitro* models and translating this to treatment success in a clinical setting is often hindered by the limitations of *in vitro* models. There is a growing need to develop improved biofilm models^20^ and developing easy-to-use solutions/models to address this problem was one of the targets of the current study. Previously, Lorenz et al. developed an *in vitro* single-species bacterial biofilm model using electrospun (ES) gelatin-glucose matrices (Gel-Gluc) as a substrate for bacteria.^21^ This model was proven to be well-suited for biofilm growth and the testing of local wound dressings. Therefore, we aimed to modify the model further and prepare a dual-species wound biofilm model using a similar biofilm model setup and testing methods, but growing more than one bacterial strain in the biofilm. This kind of complex dual-species system provides additional information about the synergistic and antagonistic behavior of bacteria, which may be present in real wound biofilms, and allows for the investigation of the efficacy of ES wound dressings in even more biorelevant conditions. Like all *in vitro* biofilm models, the disadvantage of the model is the oversimplification of the in vivo environment, as there is no host immune response present.^13^ However, the increased biorelevance of our dual-species biofilm model allows for improved simulation of bacterial interactions encountered in clinical wound settings, thereby supporting a more predictive evaluation of local antibacterial strategies.

The aim of this study was to develop a dual-species *in vitro* biofilm model that mimics the infected chronic wound environment, allowing for the efficient analysis of antimicrobial wound dressings. The biofilm was grown on Gel-Gluc, mimicking skin (referred to as artificial skin), using *S. aureus*, *E. coli*, and *P. aeruginosa* in paired combinations. The model’s suitability was validated using previously developed ES fibrous dressings containing chloramphenicol (CAM) and ciprofloxacin (CIP).

## 2 Methods

### 2.1 Materials

#### 2.1.1 Bacterial Strains, Growth Conditions, and Media Used

Three different pathogenic bacteria isolated from wounds were used for the dual-species *in vitro* biofilm model experiments: *S. aureus* DSM 2569 (ATCC 29213) (SA), *P. aeruginosa* DSM 1117 (ATCC 27853) (PA), and *E. coli* DSM 1103 (ATCC 25922) (EC). All bacterial strains were purchased from the Leibniz Institute DSMZ-German Collection of Microorganisms and Cell Cultures (Braunschweig, Germany) and stored in glycerol stocks at −80 °C.

Before the experiments, the bacteria were plated on BD Difco Lennox lysogeny broth (LB) (Becton, Dickinson and Company, Le Pont de Claix, France) agar plates and incubated overnight at 37 °C. Afterward, the plates were stored at 2–8 °C. Colonies from the plates were used for up to 1 week after plating. Bacteria for experiments were prepared in two ways: overnight liquid culture or dispersion from a solid culture. Bacterial dispersion from solid culture was prepared by collecting bacteria from LB agar plate and dispersing them in 1 mL of 1× phosphate-buffered saline (PBS). For overnight liquid culture, one colony of bacteria was suspended in 3 mL of LB and incubated overnight at 200 rpm at 37 °C. Subsequently, 1 mL of the suspension was pelleted using an Eppendorf Centrifuge 5424 R (Eppendorf AG, Germany) at 3000 rcf for 5 min and resuspended in PBS. In both cases, the resulting bacterial dispersion was normalized to approximately 10^8^ CFU per mL by diluting it to an optical density (OD_600 nm_) of 0.1 in PBS. 100 μL of the expected 10^3^ CFU/mL dispersion (10^5^ × dilution of the initially prepared 10^8^ CFU/mL dispersion) was plated on LB agar plates to determine the exact bacterial concentration. CFU counting was performed after incubation at 37 °C overnight.

Growth medium used in the biofilm studies was Dulbecco’s Modified Eagle Medium DMEM/F-12 (Sigma-Aldrich, Gillingham, United Kingdom), without l-glutamine and phenol red, together with 10% (v/v) heat-inactivated fetal bovine serum (FBS, Sigma-Aldrich, São Paulo, Brazil).

Mueller-Hinton broth (MHB, Sigma-Aldrich) was used to determine the minimal inhibitory concentration (MIC). To separate different bacteria in dual-species biofilms for CFU counting, *S. aureus* selective agar 63567-500G-F Mannitol Salt Phenol Red Agar (NutriSelect Basic, Merck KGaA, Darmstadt, Germany) was used, as suggested by the European Pharmacopoeia (2.6.13). To differentiate *P. aeruginosa* and *E. coli* from each other, Tergitol-7 agar (without triphenyl tetrazolium chloride) from Oxoid Ltd. (England) was used. The reference detection method for *E. coli* (coliform bacteria) was obtained from ISO 9308-1:2000.

### 2.2 Methods

#### 2.2.1 Preparation and Characterization of ES Fibrous Wound Dressings

Two previously prepared and characterized polycaprolactone (PCL) fibrous dressing formulations were selected to validate the dual-species *in vitro* biofilm model in the present study. The PCL-CAM fibrous dressing was prepared as described previously by Preem et al.^22^ The PCL-CIP fibrous dressings were prepared as described by Zupančič et al., with slight modifications.^23^ Briefly, CIP in its base form was used in the dressings in this study instead of CIP hydrochloride, which was used by Zupančič et al.^23^ 15% (w/w) PCL was dissolved in a 3:1 (w/w) mixture of acetic acid and formic acid and stirred overnight at room temperature (RT). Then, 5% (w/w solid-state) of CIP was added to the mixture and stirred for 1 h before electrospinning (ES). ES was conducted using a voltage of 17.2 kV, a distance from the roller of 15 cm, and a flow rate 1 mL/h. A 23G needle was used, and the roller was spinning at 40 rpm. The temperature and relative humidity (RH) were 21.3 °C and 44.8%, respectively. Unlike the previous study, the dressing was γ-sterilized. The solid-state characterization of PCL-CAM and PCL-CIP was performed. Exact descriptions of the preparation and characterization processes can be found in the Supporting Information.

#### 2.2.2 Preparation of ES Gel-Gluc

ES cross-linked Gel-Gluc was used as a substrate (artificial skin) for biofilm formation and prepared as described previously by Lorenz et al.^21^ The exact preparation of ES Gel-Gluc can be found in the Supporting Information.

#### 2.2.3 Sterilization of ES Fibrous Dressings and Other Materials Used for *In Vitro* Dual-Species Biofilm Model

All ES fibrous wound dressings and Gel-Gluc were sterilized using a 28.0–31.1 kGy dose of γ-irradiation, performed by Ionisos Baltics OÜ (Estonia). All antibacterial and antibiofilm experiments were conducted using sterilized ES fibrous dressings. Filter paper discs for the biofilm model experiments were sterilized in an autoclave at 121 °C for 15 min. DMEM/F-12 medium was sterilized by filtration through a 0.22 μm pore-size cellulose acetate filter prior to the experiment.

#### 2.2.4 Minimal Inhibitory Concentration Determination

The minimal inhibitory concentration was determined according to European Committee on Antimicrobial Susceptibility Testing (EUCAST) guidelines using the 2-fold microdilution method. Briefly, an overnight bacterial suspension (*S. aureus*, *E. coli*, *P. aeruginosa*) was diluted 10-fold into Müller-Hinton broth (MHB) and incubated for 1 h. After this, the bacteria were diluted to approximately 5 × 10^6^ CFU/mL according to the OD_600_. Antibiotic solutions (CAM, CIP) were put onto the 96-well plates and diluted 2-fold in each row using MHB. The bacterial suspension was inoculated into each well with an antibiotic solution. The well plate was incubated overnight at 37 °C in an incubator. The MICs were determined by visual inspection; the first well with no visible bacterial growth was determined as the MIC. The determined MICs were comparable to those in the EUCAST MIC database.

#### 2.2.5 Dual-Species *In Vitro* Biofilm Model

The dual-species *in vitro* biofilm model was prepared in sterile, nontreated 24-well plates (VWR International, LLC, Shanghai, China). Three sterile filter paper discs, cut to fit the size of the well, were placed at the bottom of each well. To provide a moist, wound-like environment, 250 μL of medium (DMEM/F-12 + 10% FBS) was added. Sterile 1 × 1 cm ES Gel-Gluc substrates were used for biofilm formation. These Gel-Gluc substrates were placed on top of the moist filter paper discs. Selected pathogenic bacteria were inoculated directly onto the Gel-Gluc by dropping 10 μL of bacterial suspension onto the matrix. Concentrations of 10^5^ CFU/mL for *P. aeruginosa* and *E. coli*, and 10^6^ CFU/mL for *S. aureus* were used, which means that an initial inoculum of approximately 10^3^–10^4^ CFU/matrix was applied. Bacterial suspensions from static solid cultures were prepared right before setting up the biofilm model, as described previously. For the dual-species biofilm, a suspension containing two bacterial species was prepared just prior to setting up the test. Single-species bacterial biofilms (each tested bacterial strain) were also established for control purposes by using the same inocula. Biofilms were incubated for 24 or 48 h at 37 °C. Parafilm and zip-lock bags were used to close the 24-well plates tightly to prevent evaporation from wells.

#### 2.2.6 Dual-Species *In Vitro* Biofilm Prevention and Treatment Assays

In previous studies, biofilm prevention has been assessed as the ability of a material or substance to inhibit biofilm formation,^17,24^ while biofilm treatment has been assessed as the ability to eradicate an established biofilm.^12,14,25^ Typically, biofilm research investigates either prevention or treatment alone, without integrating both approaches when assessing the efficacy of a given material or agent. In this study, we conducted both dual-species biofilm prevention and treatment assays, as described previously by Lorenz et al. for single-species assays.^21^

The prepared dual-species *in vitro* biofilm model was validated using ES fibrous wound dressings PCL-CAM, PCL-CIP, and their pristine analogs. The dressings were cut into 1 × 1 cm pieces and weighed before the assay. The model was tested in two different assays, namely, prevention and treatment, where the ES fibrous wound dressing was applied at different time points. In the *prevention assay*, the biofilm was prepared as described before, and ES fibrous wound dressings were placed on top of Gel-Gluc immediately after inoculation with bacteria. The 24-well plate was then incubated for 24 or 48 h at 37 °C. After these time points, the fibrous wound dressings and Gel-Gluc were removed from the wells, placed into 1 mL of PBS separately, homogenized, and plated for CFU counting as described earlier. For the *treatment assay*, the biofilm was prepared and incubated for 24 h at 37 °C before the ES fibrous dressings were placed on top of the Gel-Gluc. After this, biofilm incubation under the same parameters was continued for 24 h. Biofilm disruption was conducted similarly to the prevention assay. In both assays, control solutions consisting of 10 μL of antibiotic solution with a similar drug content compared to the ES fibrous wound dressings were added onto separate biofilms for comparison. In biofilm treatment assays, the homogenized bacterial suspensions were washed twice with PBS before plating to remove residues of CIP that interfered with colony counting. For this, the suspension was centrifuged at 1000 rcf for 5 min; then, 900 μL of the supernatant was discarded, replaced with PBS, and resuspended.

#### 2.2.7 Biofilm Visualization via Confocal Microscopy

Biofilms on Gel-Gluc were visualized using confocal fluorescence microscopy (CFM, LSM710, Carl Zeiss, Germany) at both analyzed time points (24 and 48 h). For this, Gel-Gluc with biofilms was removed from the well after incubation. The biofilm was fixed with a 4% formaldehyde solution in PBS. 1% (0.5 μM) stain in PBS was prepared, and 5–20 μL of the diluted stain was used per sample. The visualization of bacteria was performed using the nucleic acid stain Syto 9 (Invitrogen, Eugene, USA), with an excitation laser at 488 nm and emission collected in the 503–542 nm range. Gel-Gluc was visualized by autofluorescence emission at 410 to 502 nm, using a 405 nm laser for excitation.

#### 2.2.8 Biofilm Visualization via Electron Microscopy

Scanning electron microscopy (SEM, Zeiss EVO 15 MA, Germany) was used to visualize the surface of 48-h incubated samples, as shown previously.^21^ After incubation, samples were fixed with 4% formaldehyde in PBS for 15 min and subsequently rinsed with PBS. Dehydration was carried out using a graded ethanol series (30%, 60%, and 96%), with 5 min at each step. The samples were then air-dried overnight at RT. Before SEM analysis, the samples were mounted on aluminum stubs using carbon tape and sputter-coated with a 3 nm platinum layer in an argon atmosphere.

#### 2.2.9 Biofilm Disruption for Quantification of Viable Bacteria

Sonication and homogenization were tested to determine the most suitable method for biofilm disruption. Biofilms were pregrown on Gel-Gluc for 48 h at 37 °C, and sonication (with vortexing) or homogenization was performed to disrupt the biofilm and release the biofilm bacteria. Disruption by sonication (Bandelin Sonorex Digital 10 P) was performed for 30 s at 20% of the maximum power, followed by vortexing (Vortex-Genie 2, Scientific Industries) for 30 s; both steps were repeated 6 times. Disruption by homogenization (Bertin Technologies Precellys Evolution Touch, 2 mL tubes, 1.4 mm CK beads) was performed at 8000 rpm for 20 s. The number of biofilm bacteria was determined by CFU plating after biofilm disruption. Moreover, the two methods were compared in terms of wound dressing efficacy testing, and the method that recovered bacteria efficiently was selected. Furthermore, rinsing was tested; the studied biofilm on Gel-Gluc was placed in cold PBS and vortexed for a moment. After this, the sample was transferred into fresh PBS for further analysis. Since the rinsed and nonrinsed biofilm results were comparable, rinsing was not used.

##### 2.2.9.1 Homogenization Effects on Bacteria

The effect of homogenization on viability was assessed for all bacterial strains used in the experiments, namely *S. aureus*, *E. coli*, and *P. aeruginosa*. Bacterial suspensions with a concentration of 10^7^ CFU/mL were prepared in homogenization tubes. 1 × 1 cm^2^ Gel-Gluc matrix pieces were added to the tubes. Homogenization cycles of 10,000 rpm for 1x 20 s, 8000 rpm for 1x 20 s, 8000 rpm for 2x 20 s, and 6000 rpm for 1x 30 s were performed. Bacterial suspensions were serially diluted (10-fold) and plated out before homogenization as controls and after each homogenization cycle. CFU counting was performed, and the viability of bacteria was assessed. The test was conducted in three technical replicates for each homogenization cycle and bacterial strain. For CFU counting, 10-fold dilutions were prepared and plated on LB and selective agar plates.

#### 2.2.10 Data Analysis and Statistics

All experiments with bacteria were performed in three technical replicates, repeated three times unless stated otherwise. Other experiments were performed in three replicates. Data are presented as the arithmetic mean with standard deviation (SD), unless otherwise stated. Data analysis and visualization were performed using GraphPad Prism 10.4.1 and Microsoft Excel. Statistics were calculated, using one-way ANOVA combined with a *t*-test and Tukey or Šiidák tests. Illustrations were created with Biorender.com and Microsoft PowerPoint. Grammarly and ChatGPT-4.0 were used in writing to improve the grammar and readability of the text.

## 3 Results and Discussion

### 3.1 Dual-Species *In Vitro* Biofilm Models for Understanding Antibacterial Efficacy

It has been found that most wound infections are caused by different bacteria, and not just one bacterium is responsible for the nonhealing of the wound.^2^ Therefore, this should be kept in mind when developing and testing products for wound care. Hence, we developed a dual-species *in vitro* biofilm model that could be used to test wound care products for infectious wounds. The biofilm was developed on an artificial skin-mimicking Gel-Gluc by using combinations of two pathogenic bacteria. Different dual-species combinations were made with *S. aureus*, *E. coli*, and *P. aeruginosa*. At first, different preliminary experiments were conducted to find the most suitable parameters for dual-species *in vitro* biofilm model development. Second, the biofilm model was validated using two different methods – prevention and treatment.

#### 3.1.1 Preliminary Experiments for Dual-Species *In Vitro* Biofilm Development

Various parameters may affect the formation and properties of *in vitro* biofilms. Bacteria in biofilms exhibit different phenotypic behavior, and their metabolic activity and cell division differ from those of planktonic bacteria.^26^ It has been shown and proven that the formation of biofilms is affected by the physiological state of bacteria and the nature of the inoculum.^27,28^ In addition to the genetic background of the bacteria, the environmental conditions (temperature, pH, salt concentration, relative humidity, oxygen availability, and nutrients) may affect the formation of biofilms.^29–31^ When mimicking *in vivo* wound biofilms, the conditions should resemble the environmental factors encountered in the infected wound. In this study, preculturing conditions were varied and optimized to obtain a reproducible and suitable method for the *in vitro* biofilm model. Furthermore, different analyses and materials were tested, which enabled a better understanding of the results obtained (e.g., enumeration of bacteria by CFU using various selective agars).

##### 3.1.1.1 Preparation of Bacteria and Inoculum Size Selection

It was aimed to test whether bacteria taken from aerated overnight liquid culture versus dispersion from solid culture behave differently with our selected pathogens and which preconditioning method would be more suitable for developing *in vitro* dual-species biofilms. Different inoculum concentrations and preparation methods were tested only on the *S. aureus* and *E. coli* combination. When proceeding with the *S. aureus* and *P. aeruginosa* combination, the optimized conditions were directly used, and as they yielded similar results, no further optimization was performed. The results showed no significant differences between the dual-species biofilms from bacteria grown in different cultures regarding the number of bacteria in the final biofilm grown onto and into Gel-Gluc (Figure 1A,B). Previously, it has been shown that bacteria in biofilm and planktonic states express different gene products, and there is a transition from one state to another.^32^ Therefore, static solid cultures were used further in this study to favor biofilm formation.

**Figure 1:**
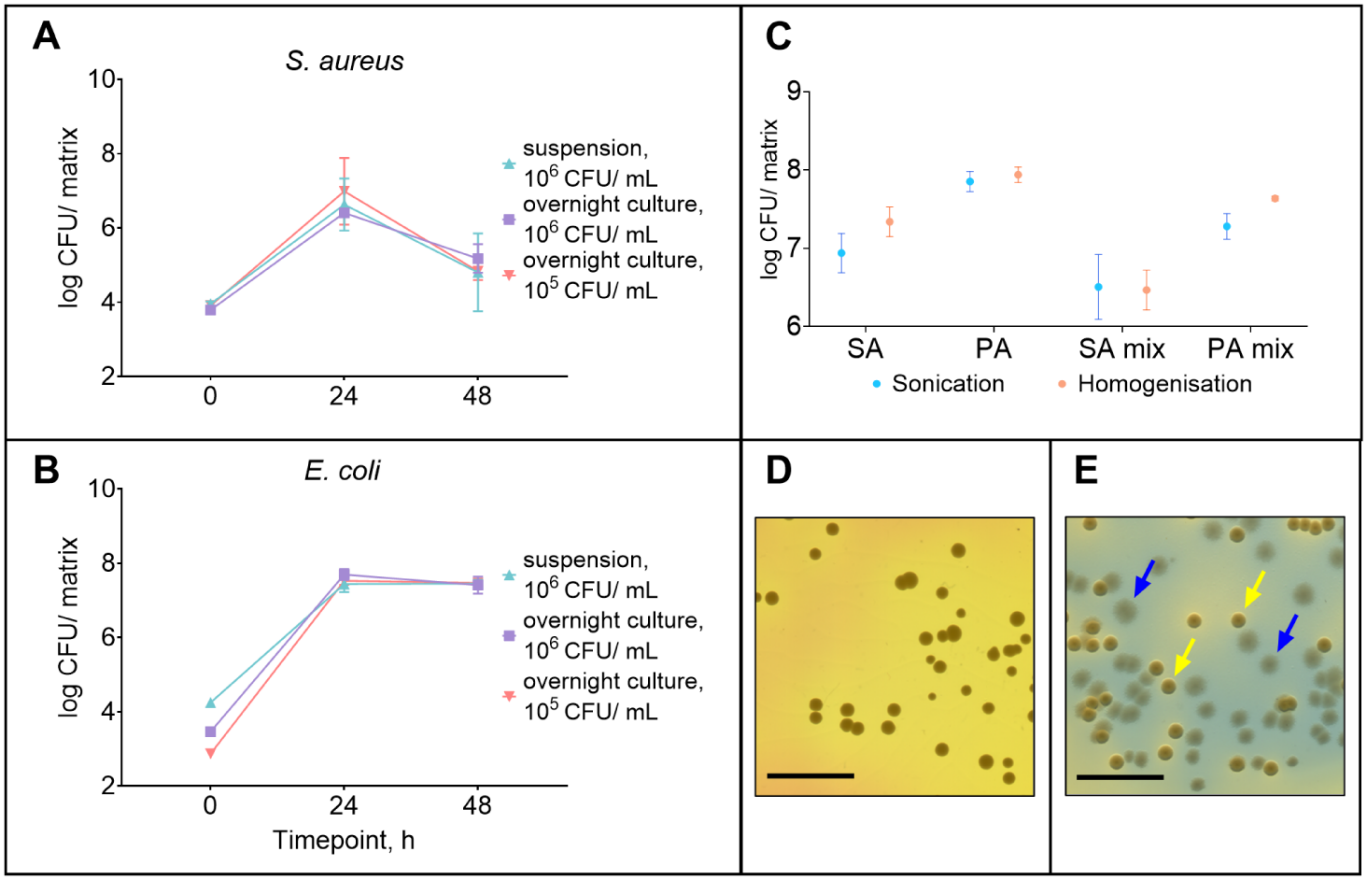
(A-B) *E. coli* and *S. aureus* dual-species biofilm growth using different preconditioning methods for *E. coli* (*N* = 3). (C). Comparison of sonication and homogenization as disruption methods on counted bacteria in *S. aureus* and *P. aeruginosa* 48-h grown biofilms (*N* = 3). (D) Images of *S. aureus* colonies on mannitol-salt agar, where the growth of Gram-negative bacteria is inhibited. (E) Images of *P. aeruginosa* and *E. coli* colonies on Tergitol-7 agar without TTC supplement, where blue arrows point to *P. aeruginosa* colonies and yellow arrows to *E. coli* colonies. Key: SA – *S. aureus*; PA – *P. aeruginosa*; SA mix and PA mix – *S. aureus* and *P. aeruginosa*, respectively, in a dual-species biofilm. *S. aureus*was prepared from the overnight liquid culture at an inoculum concentration of 10^6^ CFU/mL in all the replicates. Data are presented as the mean and SD. D-E. Scale bar: 1 cm.

Inoculum concentration testing was started with 10^6^ CFU/mL of bacterial culture for combinations of *S. aureus* and *E. coli* in a dual-species *in vitro* biofilm model (Figure 1A,B). This resulted in a lower count of *S. aureus* and domination of *E. coli* within 24 h, which has also been seen in other studies.^33,34^ It was decided to test inoculum concentrations of 10^5^ CFU/mL prepared from bacterial dispersion and overnight culture in medium, and 10^6^ CFU/mL prepared from overnight culture in medium for *E. coli*, together with 10^6^ CFU/mL for *S. aureus*, prepared from overnight liquid culture, for biofilm formation (Figure 1A,B). Based on the number of bacteria in biofilm, *E. coli* with a lower inoculum concentration (10^5^ CFU/mL) still grew into a biofilm with a similar bacterial concentration (10^7^ CFU/matrix) on the Gel-Gluc. The number of *S. aureus* was not increased significantly if a smaller *E. coli* inoculum concentration was used, but it formed a biofilm with a concentration closer to *E. coli* over 24 h. Even lower inoculum concentrations of *E. coli* (<10^5^ CFU/mL) were not used to avoid excessive variability in the results, which is known to occur.^35^ Therefore, inoculum concentrations of 10^6^ CFU/mL for *S. aureus* and 10^5^ CFU/mL for *E. coli* and *P. aeruginosa* were used for further experiments.

##### 3.1.1.2 Sonication vs Homogenization

Sonication (with vortexing) and homogenization are methods that are often used for biofilm disruption. While sonication is commonly employed for biofilm disruption and separation from solid surfaces like plastic and glass, homogenization is often used for soft materials.^36^ The exact method chosen depends on the biofilm model and the material to which the biofilm attaches. In this study, biofilms were grown into the Gel-Gluc substrate, which acts as artificial skin, and both sonication and homogenization could potentially be used. Therefore, these methods were compared to determine which is best suited for biofilm disruption. Disruption methods were compared in one biological replicate and three technical replicates to minimize the effect of time-to-time variability. In general, homogenized biofilm samples contained more bacteria than samples subjected only to sonication and vortexing (Figure 1C). For *P. aeruginosa* in dual-species biofilms with *S. aureus*, the number of bacteria detected in homogenized samples was approximately twice as high as that detected by using other disruption methods. This indicates that the sonication and vortexing combination does not extract all the bacteria from the biofilm. To ensure the viability of bacteria after biofilm disruption, different conditions of homogenization were tested 30 s × 6000 rpm, 1 and 2 × 20 s × 8000 rpm, 20 s × 10,000 rpm), and none of these conditions affected the final bacterial count. Homogenization was selected as the preferred method for biofilm disruption.

##### 3.1.1.3 Enumeration and Identification of Different Bacteria

Different agar media for bacterial enumeration were tested to separate and identify each bacterium (see Materials and Methods). If *S. aureus* was plated with *P. aeruginosa* or *E. coli* on LB agar, the Gram-negative bacteria dominated the growth, and *S. aureus* counting was impossible. Therefore, if applicable, plating was also performed on mannitol-salt agar, as suggested by the European Pharmacopoeia, where the growth of Gram-negative bacteria was inhibited (Figure 1D). *P. aeruginosa* and *E. coli* were differentiated using Tergitol-7 agar without the TTC supplement, where *P. aeruginosa* formed colonies with a blue halo, and *E. coli* formed yellow colonies with a yellow halo (Figure 1E).

#### 3.1.2 Dual-Species *In Vitro* Biofilm Formation on Top of the Gel-Gluc

This study investigated the growth dynamics and spatial distribution of *S. aureus*, *E. coli*, and *P. aeruginosa* when cultured separately and in dual combinations on Gel-Gluc over 24 and 48 h periods. This is important to understand when preparing a reproducible dual-species *in vitro* biofilm model suitable for evaluating the effectiveness of ES fibrous wound dressings. It was possible to grow dual-species biofilms on the Gel-Gluc artificial skin matrix with all tested pathogenic bacterial combinations (Figure 2).

**Figure 2:**
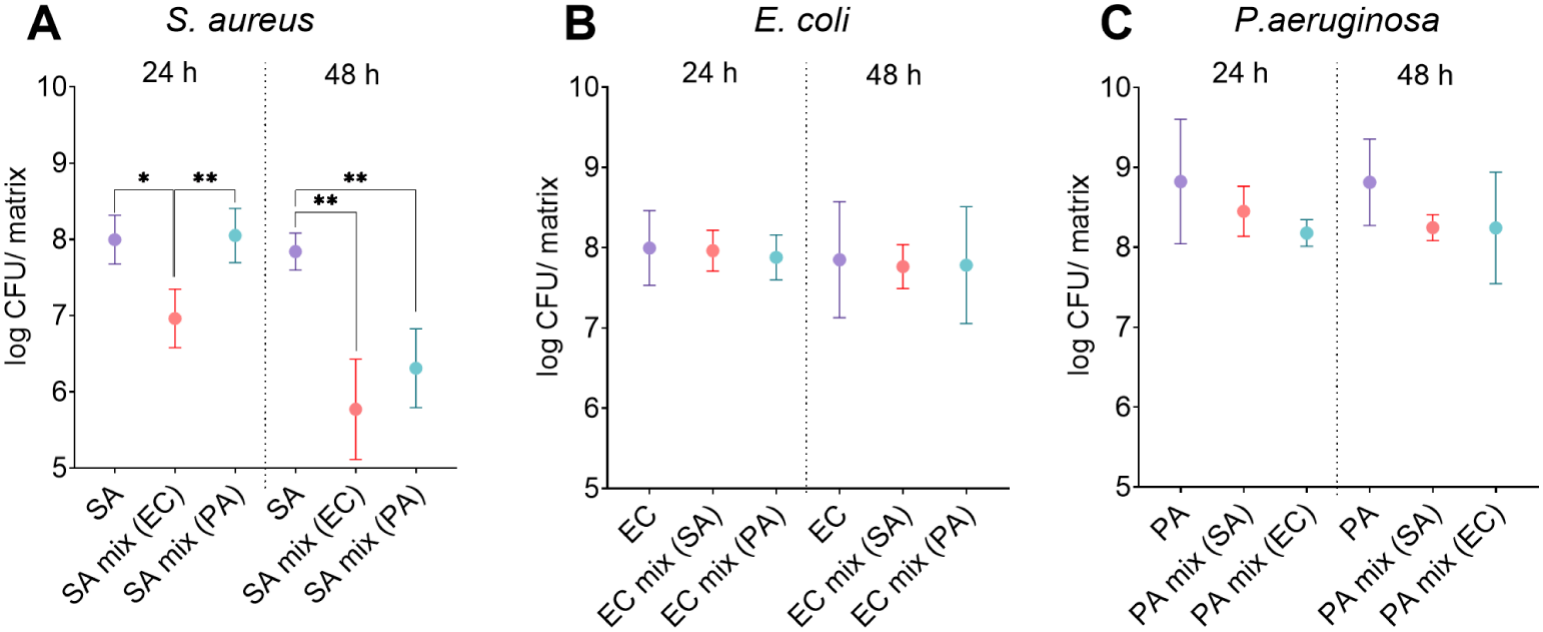
Bacterial counts of (A) *S. aureus*, (B) *E. coli*, and (C) *P. aeruginosa* in single-species and dual-species *in vitro* biofilms over 24 and 48 h. Key: SA – *S. aureus*; PA – *P. aeruginosa*; EC – *E. coli*; “mix” indicates dual-species biofilm where the coexisting bacteria are marked in brackets. Data are presented as mean and standard deviation. *p < 0.02; **p < 0.01.

In the first 24 h of incubation at 37 °C, all bacterial cultures used, whether individual or in combination, exhibited robust growth, with a recorded CFU count of approximately 10^7^–10^8^ CFU/matrix for each bacterial strain (Figure 2). It was noted that *S. aureus*, when combined with *E. coli*, grew to only approximately 10^7^ CFU/matrix, which is 10-fold less than *S. aureus* in a single-species bacterial biofilm or in combination with *P. aeruginosa*­(Figure 2A). After 48 h of incubation, the numbers of *S. aureus* had decreased to approximately 10^6^ CFU/matrix in both dual-species combinations (with *E. coli* and with *P. aeruginosa*), but not in a single-species bacterial biofilm, where the number of *S. aureus* was still approximately 10^8^ CFU/matrix, indicating that in a closed system such as this model, the Gram-negative bacteria used dominated over *S. aureus* and probably started eliminating *S. aureus* in the dual-species biofilms. It has been shown that each bacterial strain has its own specificity, and the variability from strain to strain has a great influence on the growth dynamics of bacteria.^34^ Changes in environmental conditions may largely affect the behavior—what substances they produce and how they respond to environmental changes (e.g., changed metabolism).^37^ There may be changes in the growth rate and relationships with other bacteria, which could explain some of the observed differences in CFU values.^38^

*E. coli* grew in each dual-species combination and single biofilm to similar concentrations of approximately 10^8^ CFU/matrix and remained stable over 24 and 48 h (Figure 2B). The number of *P. aeruginosa* in all the biofilm combinations (single-species and dual-species biofilms) was also similar after 24 and 48 h of incubation (Figure 2C).

It has been shown before that at the beginning of biofilm formation on glass slides, *E. coli* dominates over *S. aureus* and *P. aeruginosa* in a dual-species biofilm.^39^ In the same study, at a later time point (48 h), *P. aeruginosa* dominated the other species. Similarly, *E. coli* biofilm formation was decreased in the presence of *P. aeruginosa* on porcine skin.^40^ These findings were not seen in the present study, where three-dimensional polymeric fibrous matrix surfaces were used. It is likely that different surfaces play a role here, which is especially important at the beginning of biofilm formation (initial adsorption on the surface). *E. coli* biofilm formation was significantly different on polystyrene slides compared to porcine skin, favoring the latter.^40^ In addition, different bacterial strains were used in all compared studies, and several biofilm-forming properties may vary between different strains.^41,42^ Strain-specific traits, such as growth rate, extracellular matrix production, and secretion of different substances that influence interactions with other microbes (cooperation or competition for nutrients and space), can also influence the final biofilm population.^43–45^

Similarly, it was seen that *E. coli* dominated *S. aureus* in this study; however, this type of interaction was not seen in the *E. coli* and *P. aeruginosa* combination. In Lubbock’s biofilm model, where *S. aureus* and *P. aeruginosa* were grown together with *E. faecalis* and *Bacillus subtilis*, *S. aureus*grew at a similar rate to *P. aeruginosa*, with both reaching about the same concentrations by 24 h, and interestingly, the concentration of each pathogenic bacterium was stable over the next 24 h.^14^ A similar growth rate, where bacteria grew to form a complete biofilm, of *S. aureus* and *P. aeruginosa* was also seen in the present study. Yet, unfortunately, it was not possible to keep the concentration of *S. aureus* at the same level for as long. In a study where biofilm was grown on an ES cellulose acetate-Gel fibrous matrix, *S. aureus* and *P. aeruginosa* presented similar growth dynamics, where the concentration of both bacteria was approximately the same after 24 h, but a decrease in *S. aureus* was seen after 48 h.^15^

It has been shown that *S. aureus* survival during biofilm growth in cocultures with *P. aeruginosa* strictly depends on oxygen diffusion, where *S. aureus* survival is better with higher oxygen concentrations.^36^ This suggests that in models like those present in this study, where biofilm is grown on a liquid/air surface, the conditions would be beneficial for the growth of *S. aureus*. Furthermore, the survival of *S. aureus* in the dual-species biofilm is also increased by using bovine serum albumin (BSA) in the media instead of DMEM without any additions.^39^ In this study, FBS was added to DMEM, which contains a considerable amount of BSA and is, therefore, beneficial for the survival of *S. aureus*. The lower growth rate and higher decrease of *S. aureus* in dual-species biofilm with *E. coli* may be due to *E. coli* secretion of toxic substances. For example, it has been described that some *E. coli* strains produce the genotoxin colibactin, which could kill *S. aureus*.^33^ It must be considered that productive and metabolic properties could differ significantly from strain to strain, and specific conclusions cannot be drawn without further study into specific strains.^45^ At the same time, it has been found that a considerable number of *E. coli* strains produce bacteriocins.^46^ Yet, *E. coli* and *S. aureus* grow together in wounds. It has been discussed that in wounds, the bacteria can locate themselves at longer distances, compared to *in vitro* environments, from each other and therefore decrease the effect of substances produced by *E. coli* on *S. aureus*.^45^

Microscopic examination was performed at 24- and 48-h time points to visually identify the presence and growth of each bacterial species. *S. aureus* had a round, cocci-like shape, while *E. coli* and *P. aeruginosa* exhibited more rod-like or elliptical shapes (Figure 3).

**Figure 3:**
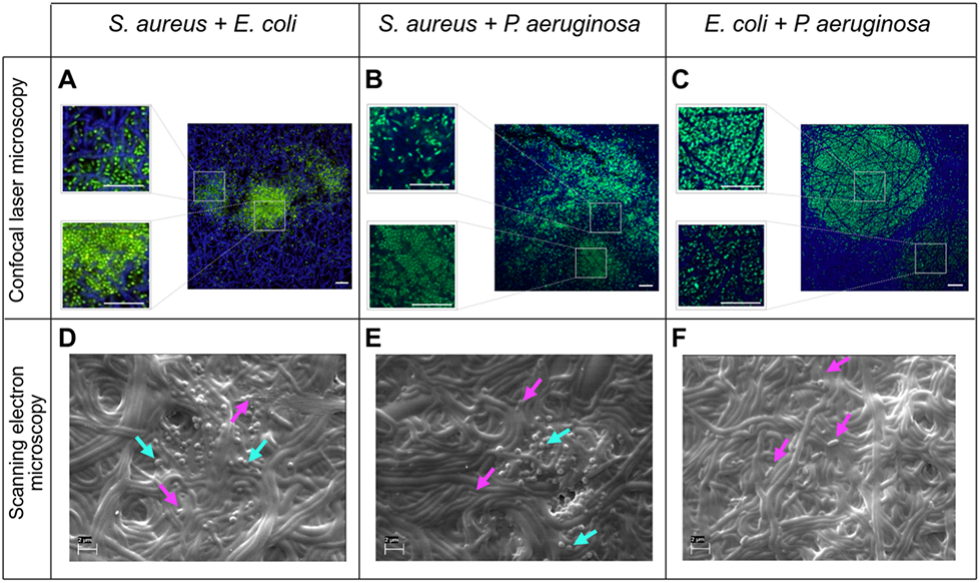
Confocal fluorescence microscopy (CFM) images and scanning electron microscopy (SEM) micrographs of dual-species *in vitro* biofilms of (A, D) *S. aureus* and *E. coli*, (B, E) *S. aureus* and *P. aeruginosa*, and (C, F) *E. coli* and *P. aeruginosa* on Gel-Gluc matrices after 48 h of incubation. CFM images are stained with Syto-9 (green), Gel-Gluc matrix autofluorescence (blue), and scale bar: 10 μm. SEM micrographs with 10× magnification have a scale bar of 2 μm. Key: cyan blue arrows - cocci shaped *S. aureus*; bright pink arrows - rod shaped bacteria (*E. coli* or *P. aeruginosa*)

While imaging the dual-species *E. coli* and *P. aeruginosa* combination biofilm, it was impossible to distinguish these bacterial species, and only a general description of their growth was provided (Figure 3C,F). When cultured individually, *E. coli* and *P. aeruginosa* demonstrated uniform growth throughout the Gel-Gluc (Figure S5). In contrast, *S. aureus* formed microcolonies within the matrix (Figure S5). These phenomena have also been described in the literature.^47^ The 48-h time point single-species biofilm results were similar to the 24-h ones, with the exception that *E. coli* and *P. aeruginosa* also formed microcolonies at 48 h, and the bacterial density was higher at 48 h compared to 24 h.

*S. aureus* growth in dual-species combinations remained similar over 24 and 48 h, forming microcolonies within the Gel-Gluc and not covering it evenly (Figures 3A–B, D–E). *E. coli* and *P. aeruginosa* covered the entire Gel-Gluc homogeneously but formed clear microcolonies as early as 24 h of incubation (Figure 3C), unlike in the 24-h single-species bacterial biofilms (Figure S5). This suggests that growing in proximity to other species induces the formation of microcolonies and could potentially promote biofilm formation, which has also been demonstrated.^36^ Furthermore, in *S. aureus* dual-species biofilms with *E. coli* or *P. aeruginosa*, it was observed that some *E. coli* or *P. aeruginosa* cells were present within or in close proximity to *S. aureus* microcolonies (Figure 3A–B, D–E), suggesting interspecies interactions or invasion within these microcolonies, which is a typical action in a resource-limited environment. The hydrodynamic conditions, nutrient concentration, bacterial motility, quorum sensing, and the actual composition of the extracellular matrix all affect the structure of biofilms.^48^ It was also seen that the formed dual-species biofilms were slightly different in every experiment, considering the density of bacterial cells over Gel-Gluc, the sizes of microcolonies, and the number of microcolonies observed. This may be related to the constantly changing environmental parameters influencing the results. For example, relative humidity, exact temperature, inoculum size, and the thickness of a single Gel-Gluc piece may have influenced the spatial location of bacteria.

#### 3.1.3 Biofilm Model Validation Using Drug-Loaded ES Fibrous Wound Dressings

In this study, sterile antibiotic-loaded ES fibrous wound dressings were used to validate the developed dual-species *in vitro* biofilm model. Previously developed and characterized fibrous wound dressings were used, namely PCL-CAM and pristine PCL (ES using a chloroform–methanol solvent system),^49^ and PCL-CIP and pristine PCL (ES using a formic acid–acetic acid solvent system), slightly modified from Zupančič et al.^23^ Different antibiotic-loaded dressings enabled the comparison of the antibacterial efficacy of the dressings with different drugs against dual-species wound infections (CAM vs CIP) and the effect of fibers with different morphologies. All fibrous dressings were successfully ES and γ-sterilized before use. Solid-state characterization confirmed that the prepared fibrous dressings were similar to those described previously (data in the Supporting Information).

PCL-CAM wound dressing was tested only on *S. aureus* and *E. coli* biofilm due to the *P. aeruginosa* resistance to CAM.^50^ PCL-CIP dressing was tested on all three dual-species biofilm combinations: *S. aureus* with *E. coli*, *S. aureus* with *P. aeruginosa*, and *E. coli* with *P. aeruginosa*. The dual-species *in vitro* biofilm models were validated using two different approaches: (i) biofilm prevention (inhibition) and (ii) biofilm treatment assays, as described previously by Lorenz et al.^21^ This enabled us to test various antibacterial wound dressings for efficacy and understand their modes of action and limitations against relevant wound pathogens. Unlike single-species biofilm models, it was possible to better understand the potential efficacy of the antibacterial wound dressings against different pathogens in a biofilm at the same time. Similarly to biofilm development experiments, 24- and 48-h time points were used for testing. The mean changes in log CFU compared to the same time point of untreated biofilm development were calculated to determine the log reduction of each sample. All the results are presented as the total number of bacteria on Gel-Gluc and ES fibrous matrices, where applicable. The ratio of bacterial numbers located on one or the other substrate is presented in Figure S6.

##### 3.1.3.1 Biofilm Prevention

The results showed that PCL-CAM fibrous dressings were effective against the formation of dual-species biofilms at 24 and 48 h in *S. aureus* and *E. coli* within a dual-species biofilm model (Figure 4D–E). In some cases, a small number of bacteria were detected even when a PCL-CAM wound dressing or CAM solution was applied. However, the number of detected bacteria was around the detection limit. No significant difference was found between the efficacy of the CAM solution and PCL-CAM fibers. In a similar experiment, where the PCL-CAM fibrous wound dressing was used against a single-bacterium biofilm, no bacterial growth was detected after 24 or 48 h.^21^

**Figure 4:**
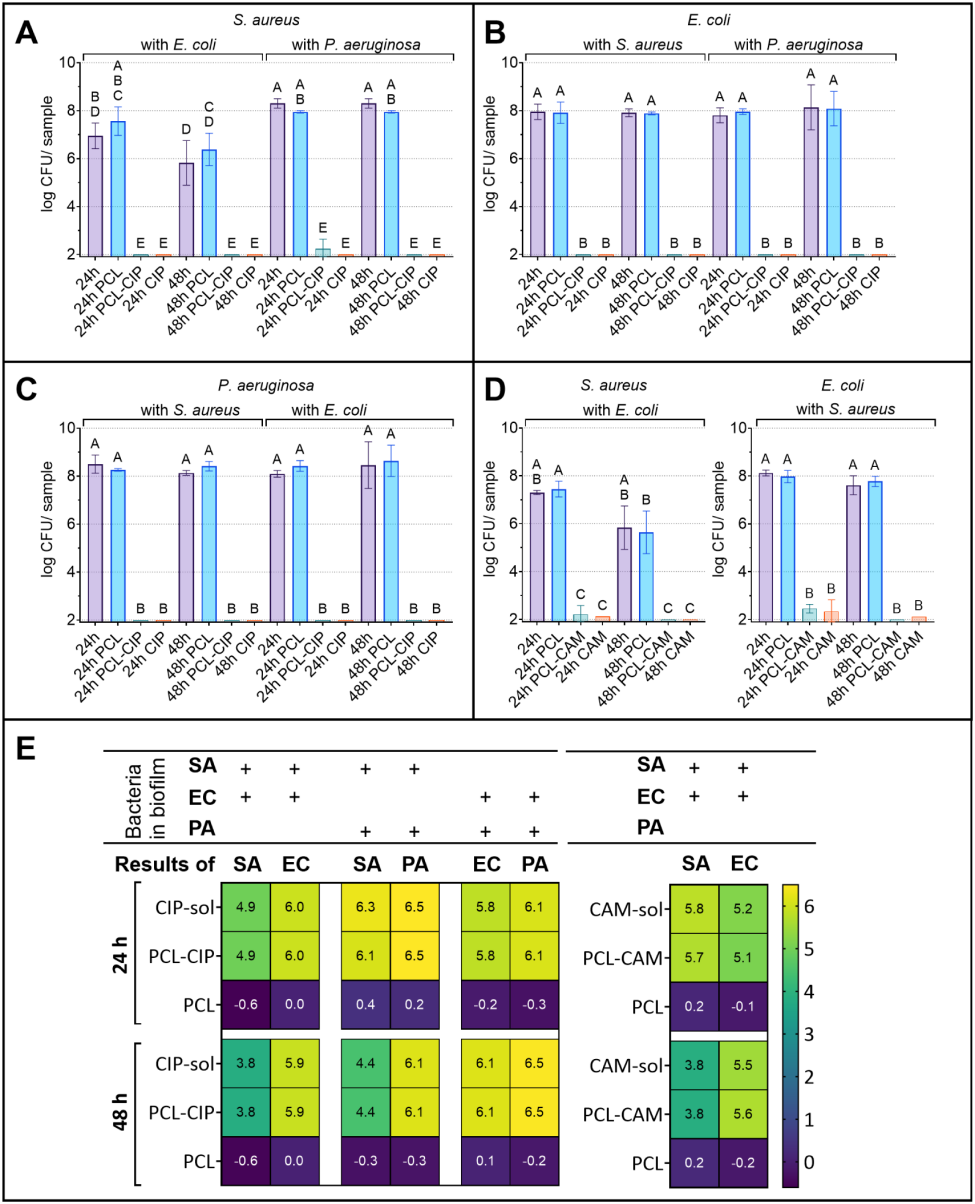
(A-C) *S. aureus*, *E. coli*, and *P. aeruginosa* CFU counts after prevention experiments in untreated samples and in samples with pristine PCL fibers, PCL-CIP, and CIP solution after 24 and 48 h in different dual-species combinations. (D) *S. aureus* and *E. coli* CFU counts after prevention experiments in untreated samples and in samples with pristine PCL fibers, PCL-CAM, and CAM solution after 24 and 48 h. (E) Log reduction of bacterial CFU after biofilm prevention compared to untreated biofilm at 24 and 48 h in different dual-species combinations. Key: PCL-CIP – polycaprolactone ciprofloxacin fibrous dressing; PCL-CAM – polycaprolactone chloramphenicol fibrous dressing; sol – solution (CIP solution for PCL-CIP and CAM solution for PCL-CAM formulation); PCL – pristine PCL-CIP or PCL-CAM fibrous dressing; SA – *S. aureus*; EC – *E. coli*; PA – *P. aeruginosa*; 24 and 48 h – untreated biofilm at marked time points. Data are presented as mean ± SD. Statistical significance was determined using one-way ANOVA with Šidák’s post hoc test, considering significant if *p* < 0.05 and presented in A–D as compact letter display, where no statistically significant differences were observed between dressings sharing a letter.

PCL-CIP fibrous wound dressings completely prevented the development of *S. aureus* and *E. coli* biofilm, as no viable bacteria were detected in the samples, in contrast to PCL-CAM fibrous wound dressing samples, which occasionally revealed low bacterial counts (Figure 4A–C, D). PCL-CIP fibrous dressing was also efficient in preventing the formation of *P. aeruginosa* and *S. aureus* biofilm, as well as *P. aeruginosa* and *E. coli* biofilm (Figure 4A–C). Again, the results were as expected; the bacterial concentration at the beginning of the experiment was low (10^3^–10^4^ CFU per matrix), and bacteria were expected to be in planktonic form. The antibiotic concentrations in the wells were high compared to the MICs, at least 20× or 365× higher for CAM and CIP, respectively (Table 1).

**Table 1:** Minimal Inhibitory Concentrations (MIC, μg/mL) of Antimicrobial Substances Used on Pathogenic Bacteria in the Dual-Species *In Vitro* Biofilm Model^a^

				calculated mean concentration in sample wells	
antibiotic	bacteria	EUCAST MIC	measured MIC	based on content analysis	based on the 24 h release test
CAM	*P. aeruginosa* DSM 1117	NA	NA	380	152
	*E. coli*	1–16	4		
	*S. aureus* DSM 2569	8–16	8		
CIP	*P. aeruginosa* DSM 1117	0.25–0.5	0.5	182.5	182.5
	*E. coli*	0.002–0.06	0.008		
	*S. aureus* DSM 2569	0.125–0.5	0.25		

a Key: CAM: chloramphenicol; CIP: ciprofloxacin; EUCAST: European Committee on Antimicrobial Susceptibility Testing; “NA” – not applicable, as MIC is not available in the EUCAST MIC database/MIC testing was not performed.

Log reductions for all prevention assays illustrate the effectiveness of tested fibrous dressings and antibiotics (Figure 4E). Furthermore, the pristine PCL fibrous dressings do not influence the dual-species biofilm formation much (Figure 4E).

##### 3.1.3.2 Biofilm Treatment

The studied ES fibrous dressings’ antibiofilm properties were assessed in biofilm treatment assays, where the dressings were applied to 24-h pregrown biofilms. After treatment of *S. aureus* and *E. coli* 24-h biofilms with PCL-CAM fibrous wound dressing or CAM solution, no significant decrease in bacterial counts was seen (Figure 5D, E). This demonstrates the bacteriostatic effect of CAM, as it is not capable of efficiently treating pregrown biofilms.^21^

**Figure 5:**
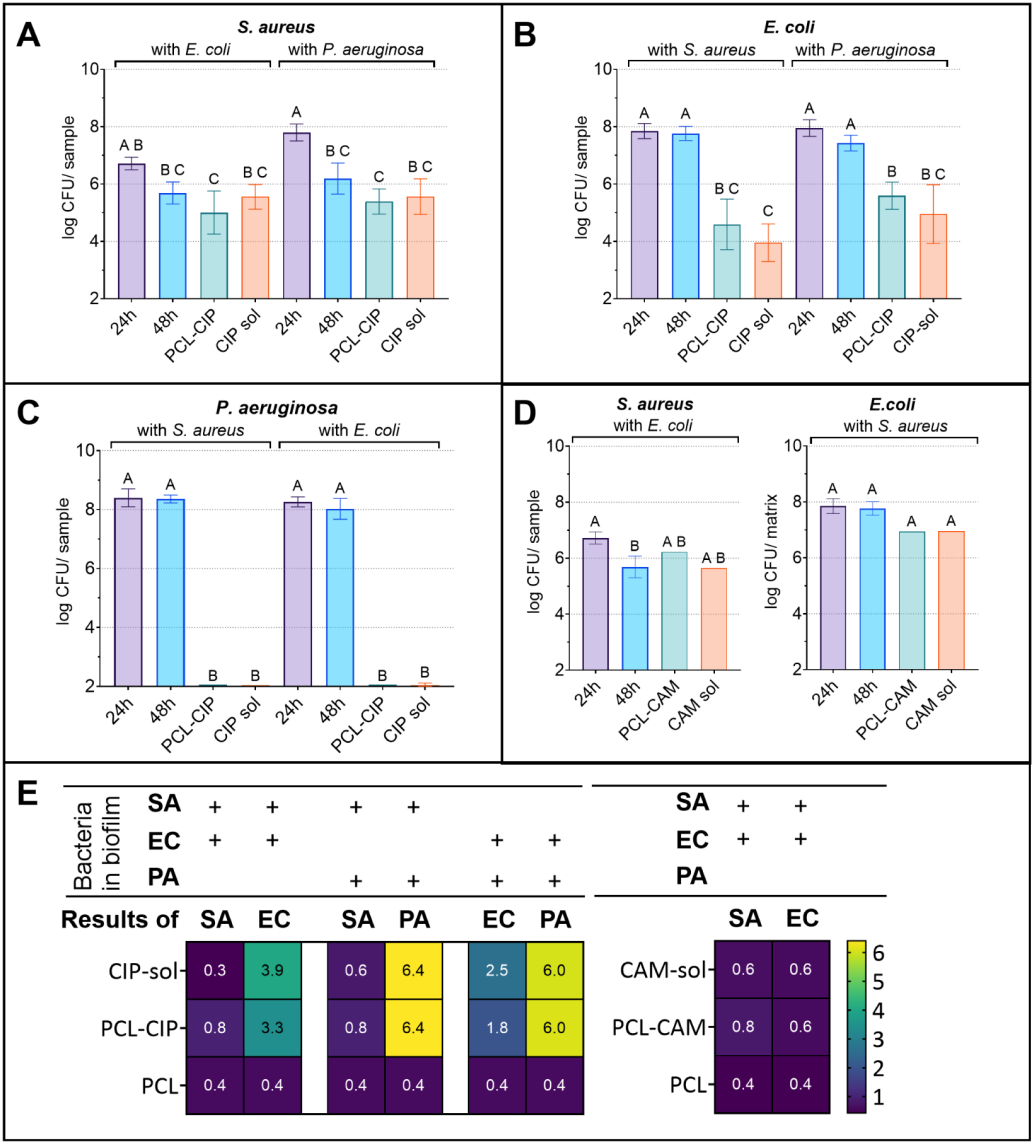
(A-C) CFU counts of each bacterium in untreated dual-species biofilms and biofilms after PCL-CIP/CIP treatment. (D) CFU counts of *S. aureus*and *E. coli* in untreated dual-species biofilms and in biofilms after PCL-CAM/CAM treatment. (E) Log reduction of bacterial CFU counts from different bacterial combinations after biofilm treatment compared to untreated biofilm at 24 h. Key: PCL-CIP – polycaprolactone ciprofloxacin fibrous dressing; PCL-CAM – polycaprolactone chloramphenicol fibrous dressing; sol – solution (CIP solution for PCL-CIP and CAM solution for PCL-CAM formulation); PCL – pristine PCL-CIP or PCL-CAM fibrous dressing; SA – *S. aureus*; EC – *E. coli*; PA – *P. aeruginosa*; 24 and 48 h – untreated biofilm at marked time points. Data are presented as mean ± SD. Statistical significance was determined using one-way ANOVA with Šidák’s post hoc test, considering significant if *p* < 0.05 and presented on (A)–(D) as compact letter display, where no statistically significant differences were observed between dressings sharing a letter.

PCL-CIP and CIP solution treatment impacted all tested dual-species biofilm combinations (Figure 5A–C, E). Yet, the number of *S. aureus* did not decrease significantly in any combination after CIP treatment (Figure 5A, E). It is essential to consider that the number of *S. aureus* after treatment was similar to that of the 48-h untreated samples. Based on this study, it is impossible to say if the decrease of *S. aureus* in biofilm treatment tests is induced by CIP or other bacteria in the dual-species biofilm. However, it has been described before that *P. aeruginosa* exoproducts may increase or decrease the resistance of *S. aureus* to CIP, but the change is dependent on the specific *P. aeruginosa* strain.^51^ Therefore, the presence of *P. aeruginosa* can potentially increase the survival of *S. aureus* and enhance the expression of virulence factors.^52^ Proof exists that both bacteria (*P. aeruginosa* and *S. aureus*) show increased survival rates to antibiotic treatment when grown together in planktonic cocultures and demonstrate enhanced antibiotic tolerance in wound models.^18^ Furthermore, a change in the *S. aureus* phenotype after CIP treatment caused the colonies to appear in different sizes (Figure 6).

**Figure 6:**
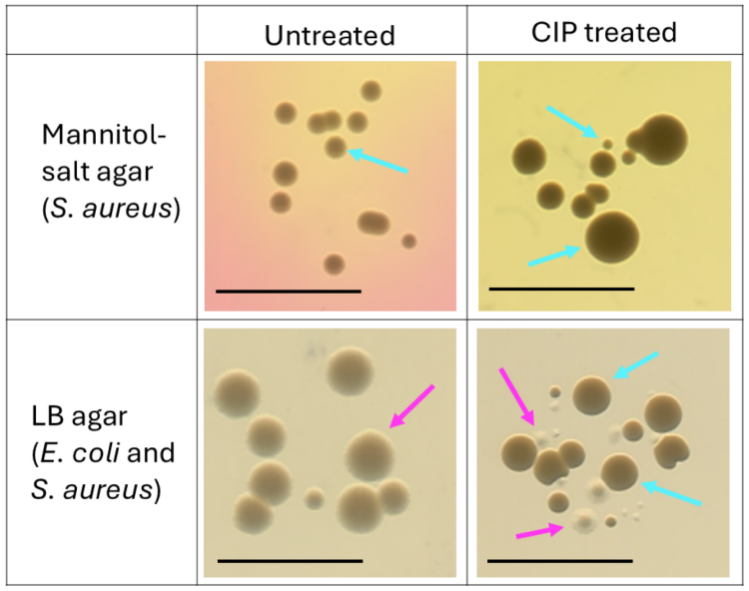
Morphology of bacterial colonies from untreated and CIP-treated dual-species biofilms of *S. aureus* and *E. coli* on *S. aureus* selective (mannitol-salt) and nonselective (LB) agars after 48 h and after treating 24 h grown dual-species biofilms with ciprofloxacin (CIP) for 24 h. On nonselective LB agar *E. coli* overgrows *S. aureus* after 24 h of incubation and no *S. aureus* colonies were detected, while with CIP treatment both bacteria can be detected on LB agar, confirming the presence of dual-species biofilms before plating. Key: cyan blue arrows – *S. aureus* colonies; bright pink arrows – *E. coli* colonies.

It has been demonstrated that exposure to antibiotics, especially fluoroquinolones, influences the phenotype of bacteria, and this could play a role in resistance development.^53^

In the case of *E. coli*, the reduction in bacterial numbers was influenced by the identity of the cocultured species. The number of *E. coli* after CIP treatment was reduced by 3.3–3.9 log units in dual-species biofilm with *S. aureus*, while in combination with *P. aeruginosa*, the log reduction was 1.8–2.5 units. Although the change is not statistically significant, it is noteworthy that the effect of antibiotics may differ in different combinations of bacteria (Figure 5A–C, E). Somewhat similarly to *S. aureus* after CIP treatment, the remaining *E. coli* that had exposure to CIP had heterogeneous phenotypes, where some colonies appeared to be smaller compared to typical colonies and/or were colorless/transparent (Figure 6). CIP treatments were effective against *P. aeruginosa* in both dual-species combinations (Figure 5C, E). It has been shown before that CIP can kill nongrowing *P. aeruginosa*.^54^ This explains the efficacy of CIP treatment in this experiment, as based on 24- and 48-h results, *P. aeruginosa* could likely be in the nongrowing phase after 24 h of biofilm development. At the same time, in another study, it was found that *S. aureus* increases *P. aeruginosa* susceptibility to CIP; interestingly, in that study, the concentration that completely eradicated *P. aeruginosa* in dual-species biofilm also killed *S. aureus*, and it was approximately 1.5 times higher compared to the present study.^55^

## 4 Conclusions

New active substances, dosage forms, and wound care products can help fight chronic wound infections and may have improved effects on healing. However, for development and testing, we need biorelevant and reliable *in vitro* efficacy testing methods that allow fast and reliable comparisons between various formulations. ES Gel-Gluc is a suitable substrate for preparing a stable dual-species *in vitro* biofilm. Homogenization, instead of sonication or vortexing enabled sufficient release of the biofilm bacteria. The developed dual-species *in vitro* biofilm model was suitable for the preliminary assessment of the antibacterial efficacy of fibrous wound dressings. Further studies will provide deeper insight into bacterial growth dynamics under various conditions, which are crucial for developing more effective strategies to prevent and treat biofilm-related infections.

## Supplementary Material



## Notes

Additional information about experimental methods and results: Materials, methods, and results of electrospun fiber preparation and characterization (PDF)
