# Supplementary material for: Preparing a Dual-Species In Vitro Biofilm Model for Testing Antibiofilm Efficacy
Source: Mol Pharm. 2025 Oct 19;22(11):6862–73. doi: 10.1021/acs.molpharmaceut.5c00798 (PMC12587443; doi:10.1021/acs.molpharmaceut.5c00798)
Supplement: Supplementary file 1 [file mp5c00798_si_001.pdf]

Supporting information for

# Preparing dual-species *in vitro* biofilm model for the testing of antibiofilm efficacy

Kelli Randmäe[a], Kairi Lorenz[a], Marta Putrinš[a], Tanel Tenson[b], Karin Kogermann[a]*

[a]Institute of Pharmacy, University of Tartu, Nooruse 1, 50411 Tartu, Estonia

[b]Institute of Technology, University of Tartu, Nooruse 1, 50411 Tartu, Estonia

**email:* karin.kogermann@ut.ee

# 1. Preparation and characterization of electrospun (ES) fibrous dressings

## 1.1 Materials and Methods

### 1.1.1 Materials

*Drugs, polymers, solvents and supplies.* Antibacterial agents chloramphenicol (CAM, PubChem CID: 5959) and ciprofloxacin (CIP, PubChem CID: 2764) were purchased from Sigma-Aldrich Inc. (Germany). Polycaprolactone (PCL) (Mn 80 000) used in this study was obtained from Sigma-Aldrich Inc. for PCL-CAM fibrous dressings and from Purac Corbion (The Netherlands) for PCL-CIP fibrous dressings. Acetic acid (AA) (99.8–100.5%, puriss p.a.) and formic acid (FA) (≥98%, puriss p.a.) were purchased from Fluka Honeywell. Chloroform (CF, puriss p.a.), methanol (MeOH, gradient grade). All solvents were of reagent grade and were used as received without further purification. For gelatin-glucose matrix (Gel-Gluc) preparation, type A gelatin from porcine skin (Gel), and anhydrous D-(+)-glucose (Gluc) were purchased from Sigma-Aldrich, AA (99.8–100.5%, puriss p.a.) was purchased from Fluka Honeywell.

### 1.1.2 Preparing electrospun (ES) fibrous wound dressings

Two previously prepared and characterized PCL fibrous dressing formulations were selected for validating the dual-species *in vitro* biofilm model in the present study. PCL-CAM fibrous dressing was prepared as described previously by Preem *et al.* [1]. Briefly, 12.5% (w/V) of PCL and 4% (w/w) CAM were dissolved in a CF:MeOH 3:1 (V/V) mixture and stirred at room temperature (RT) overnight. PCL-CIP fibrous wound dressing was prepared as described previously by Zupančič *et al.,* with slight modifications [2].

CIP in a base form was used in the dressings in this study instead of CIP hydrochloride which was used by Zupančič *et al.* Briefly, 15% (w/w) PCL was dissolved in a 3:1 (w/w) mixture of AA and FA and stirred overnight at RT, 5% (w/w solid-state) of CIP was added to the mixture and stirred for 1 h before ES. The addition of CAM and CIP was not performed when preparing control PCL fibers. All ES was performed on the ESR200RD ES system (NanoNC, Seoul, Republic of Korea) and exact ES parameters are in **Table S1.**

**Table S1.** Electrospinning (ES) parameters of prepared fibrous dressings.

| Fibrous dressing | Temperature | Relative humidity (RH) | ES voltage | Distance from collector | Flow rate | Needle | Roller spinning rate |
|---|---|---|---|---|---|---|---|
| PCL-CAM | 22.0 °C | 38 % | 9 kV | 14 cm | 1 mL/h | 25G | 20 rpm |
| PCL (control for PCL-CAM) | 21.1 °C | 28.5 % | 9 kV | 14 cm | 1 mL/h | 25G | 20 rpm |
| PCL-CIP | 21.3 | 44.8 % | 17.2 kV | 15 cm | 1 mL/h | 23G | 40 rpm |
| PCL (control for PCL-CIP) | 23.2 °C | 33 % | 17 kV | 15 cm | 1 mL/h | 23G | 40 rpm |

Key: CAM - chloramphenicol; CIP – ciprofloxacin; PCL – polycaprolactone.

### 1.1.3 Preparing ES Gel-Gluc

ES crosslinked Gel-Gluc were used as substrates (artificial skin) for biofilm formation and prepared as described previously by Lorenz *et al.* [3]. The ES solution was prepared by dissolving 25% (w/V) Gel and 10% (w/w solid-state) Gluc in 10 M AA. ES was performed using a flow rate of 0.9 mL/ h, a needle of 23 G, and a voltage of 18 kV at RT (22 ± 2 °C and ~37% RH). Gel-Gluc were heat cross-linked at 170 °C for 3 h in a heating chamber, which is described in more detail previously by Siimon *et al.* [4,5]. These ES Gel-Gluc have been studied and fully characterized (morphology, solid-state properties, thickness) in previous studies [4,5,3,6].

### 1.1.4 Characterization of ES fibrous wound dressings

*Morphology analysis.* Prepared ES fibers were examined under scanning electron microscopy (SEM, Zeiss EVO 15 MA, Germany) for the morphology and fiber diameters. Randomly selected areas of the fibrous dressings were mounted on aluminum stubs and magnetron-sputter coated with a 3 nm platinum layer in an argon atmosphere before microscopy. For fiber diameter and size distribution measurements, 10 000 x magnification was used. For SEM measurements, 100 fibers, or as many as possible, were measured from three SEM micrographs. Data are presented as an arithmetic mean with standard deviation (SD).

*Drug content analysis in ES fibrous dressings.* The drug content within ES fibrous dressings was analyzed using high-performance liquid chromatography (HPLC). ES drug-loaded fibrous dressings were cut into 1 $cm^2$ pieces and weighed. The pieces of PCL-CAM dressing were dissolved in CF:MeOH (3:1 V/V) mixture. HPLC was performed according to the official European Pharmacopoeia method for a related substance CAM sodium succinate. The mobile phase used was 20 g/L solution of phosphoric acid R, methanol R, and water R (5:40:55 V/V/V). The flow rate was 1.0 mL/min, and the injection volume was 20 μL. The detection wavelength was 275 nm.

1 $cm^2$ pieces of PCL-CIP fibrous dressings were dissolved in 1 mL of AA: FA mixture (3:1 m/m), and 4 mL of water was added to the solution to sediment the PCL. Sedimented PCL was removed by filtration through filters with a pore size of 45 μm. HPLC was performed, using a mobile phase containing acetonitrile R, 2.45 g/ L solution of phosphoric acid R adjusted to pH 3.0 with triethylamine R (87:13 V/V). HPLC analyses were performed using Shimadzu Prominence LC20 with a PDA detector equipped with a column Phenomenex Luna C18(2), 250 × 4.6 mm, 5 mm.

*Drug release analysis. In vitro* drug release from the prepared PCL-CIP fibrous dressing was carried out in 1x phosphate buffered saline (PBS, pH 7.4) at 37 °C in a static solution mimicking the wound environment. 1 $cm^2$ fibrous dressing pieces were used. 200 μL samples were taken from the dissolution environment at 5 min, 1 h, 4 h, 24 h, 48 h, and 72 h time points. The volume was replaced with the same amount of PBS. Samples were analyzed using HPLC, as described above.

*Solid state analyses of PCL-CIP fibrous dressings.* PCL-CIP fibrous dressing, its raw materials, and their physical mixture (PM) were further characterized using X-ray Diffraction (XRD) and Attenuated Total Reflection Fourier Transformed Infrared (ATR-FTIR) Spectroscopy. The XRD experiments were carried out using X-ray diffractometer (D8 Advance, Bruker AXS GmbH, Germany) in a symmetrical reflection mode (Bragg–Brentano geometry) with CuKa radiation (1.54 Å). The scattered intensities were measured with the LynxEye one-dimensional detector, which included 165 channels. The angular range measured for diffractograms was from 5° to 40° 2-θ with the step size of 0.0184° 2-θ. ATR-FTIR spectroscopy was performed using an IR Prestige-21 spectrophotometer (Shimadzu Corp., Kyoto, Japan) with a Specac Golden Gate Single Reflection ATR crystal (Specac Ltd., Orpington, UK). The spectra were collected between 600 and 4000 $cm^{-1}$, and 60 scans were performed for each sample.

Raman scattering microspectroscopy (RSM) was performed using a Reinshaw InVia micro Raman spectrometer (Reinshaw, England) with a CCD Camera (1040 × 256) and 633 nm diode laser excitation. The exposure time of 120 s and a 50× objective were used for the measurements. Raman data were collected in the spectral range of 50 to 4000 $cm^{-1}$ with 1 $cm^{-1}$ resolution.

# 2. Results and discussion

## 1.2 Characterization of ES fibrous wound dressings

In this study, sterile antibiotic-loaded ES fibrous wound dressings were used to validate the developed dual-species bacterial *in vitro* biofilm model. Previously developed and characterized fibrous wound dressings were used, namely PCL-CAM and pristine PCL (ES using CF-MeOH solvent system) [1], and PCL-CIP and pristine PCL (ES using AA:FA solvent system), slightly modified from [2].

Different antibiotic-loaded dressings enabled the comparison of the antibacterial efficacy of the dressings with different drugs against dual-species bacterial infections (CAM *vs* CIP) and the effect of fibers with different morphologies. All fibrous dressings were successfully ES and γ-sterilized before use. Different analyses were conducted to assess and understand their physicochemical properties and antibacterial characteristics.

### 1.2.2 Morphology

The morphology analysis of ES fibrous dressings was performed before and after γ-sterilization, and it was confirmed that neither morphology nor fiber diameter changed due to γ-sterilization (**Figures S1, S2**). Hence, only γ-sterilized fibrous dressings were selected to further characterization and used in *in vitro* biofilm models for testing.

The mean diameter of prepared PCL-CAM fibers was 0.650 ± 0.337 μm, whereas the respective pristine PCL fibers had a mean diameter of 0.691 ± 0.409 μm (**Figures S2A** *vs* **S2B**). The size distribution histograms revealed that both pristine and CAM-loaded fibers have fibers with different sizes (below and above micrometers). It was seen that the addition of CAM into the fibers did not noticeably affect the fiber diameter nor its size distribution. Preem *et al.* previously described a similar result, but their measured fibers' diameters were slightly thinner, and blank PCL fibers were more homogeneous compared to the results of the present study [1]. The difference could be explained by the use of different PCL batches, which could contain polymers with slightly different molecular weights.

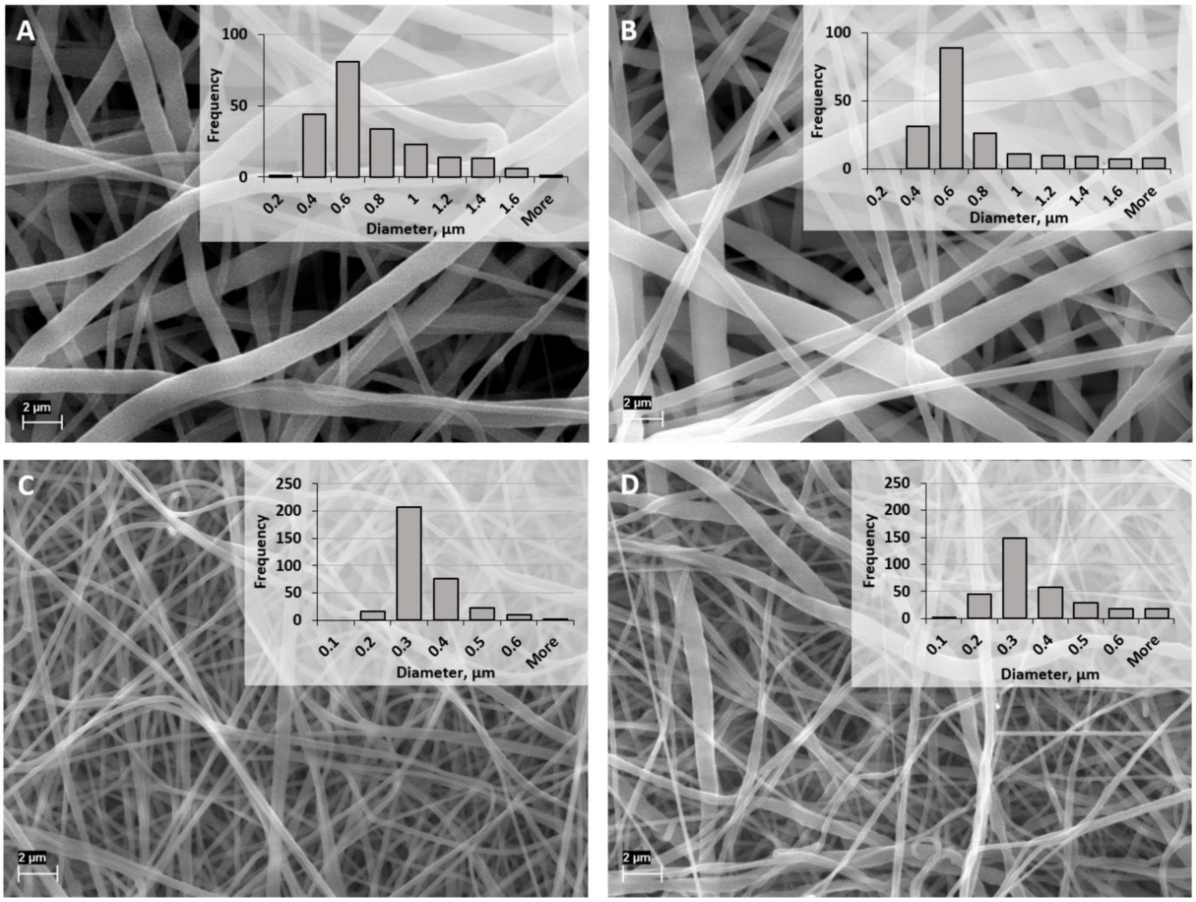


**Figure S1.** SEM micrographs and histograms of non-sterilized ES A. PCL-CAM fibers and B. their pristine PCL control fibers, C. PCL-CIP fibers and D. their pristine PCL control fibers (D) (minimum N=200).

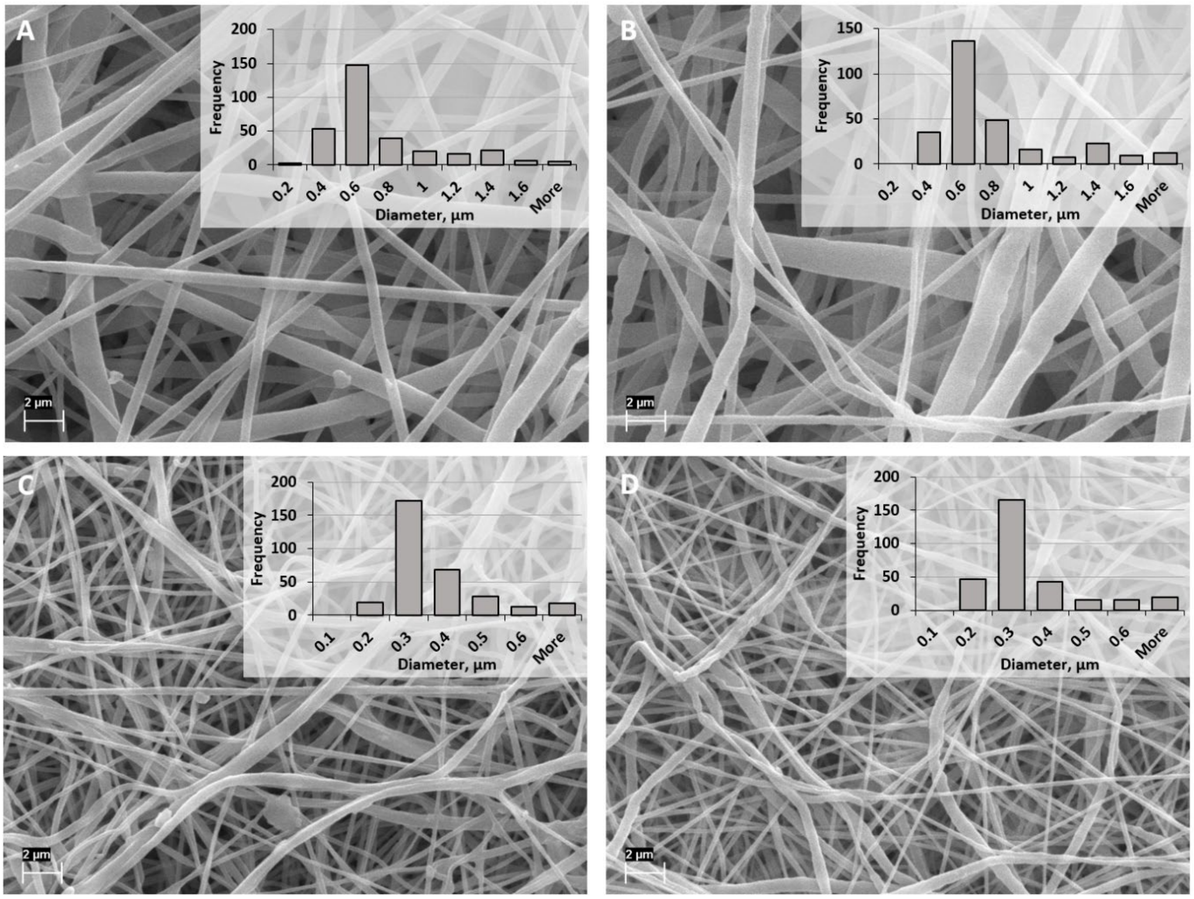


**Figure S2.** SEM micrographs and histograms of γ-sterilized ES A. PCL-CAM fibers and B. their pristine PCL control fibers, C. PCL-CIP fibers and D. their pristine PCL control fibers (N=300).

ES of pristine PCL fibers from AA:FA solution (control for PCL-CIP) was successful and resulted in smooth fibers. Whilst SEM micrographs and histograms showed that PCL fibers containing CIP were smoother, and the size of fibers was more homogenous than for pristine PCL fibers (**Figures S2C** *vs* **S2D**). The mean diameter of PCL-CIP fibers was 0.294 ± 0.078 µm, and for the pristine PCL fibers, 0.319 ± 0.156 µm. PCL fibers prepared in this study were thinner and more homogeneous compared to a previous study by Zupančič *et al.*, where similar ES fibers containing CIP-HCL instead of CIP base were prepared [2]. The size and distribution of PCL-CIP fibers remained similar to those prepared in the previous study [2]. Compared to PCL-CAM fibers and their control PCL fibers (ES with CF:MeOH), PCL-CIP fibers and their control fibers (ES with AA:FA) were more uniform and homogeneous.

### 1.2.3 Drug content analysis within ES fibrous dressings

The drug content of prepared fibrous dressings was measured using HPLC after the ES of the fibers and γ-sterilization. It has previously been shown that γ-sterilization may cause some degradation of active ingredients [7]. The results of the content analysis are presented in **Table S2**.

**Table S2.** Stability of chloramphenicol (CAM) and ciprofloxacin (CIP) in fibrous dressings after electrospinning (ES) and γ-sterilization.

| Formulation | Calculated drug content in fibrous dressings | Drug content in fibrous dressings after ES | Drug content in fibrous dressings after γ-sterilization |
|---|---|---|---|
| PCL-CAM | 4.00 % | 4.00 ± 0.08% | 3.90 ± 0.17 % |
| PCL-CIP | 4.90 % | 4.09 ± 0.11 % | 3.33 ± 0.03 % |

Key: CAM – chloramphenicol; CIP – ciprofloxacin, ES – electrospinning; PCL – polycaprolactone.

Content analysis revealed that CAM was stable in PCL-CAM fibers during the ES, but its concentration decreased after γ-sterilization as described before [1,7].

The CIP content in PCL-CIP fibers decreased both during ES and after γ-sterilization. It has been shown before that the ES process and γ-sterilization may degrade chemical substances, including active pharmaceutical ingredients (APIs), as these processes may involve harsh solvents, high voltage, and/or radiation [7]. Yet, there are reports informing that CIP does not degrade during the ES process [8]. Unfortunately, no direct comparisons can be made as different solvents and CIP solid state form were used and no sterilization was conducted for PCL-CIP fibers by Uhljar *et al* [8].

### 1.2.4 Solid state characterization of ES fibrous dressings

PCL-CAM fibrous dressings have been fully characterized (both γ-sterilized and non-sterilized) [1,7]. Therefore, the solid-state characterization results of γ-sterilized PCL-CIP fibrous dressings are presented in the present study, although the drug content and morphology analyses were performed for all different ES fibrous dressings prepared.

*XRD analysis.* The XRD patterns of raw CIP powder exhibited crystalline characteristics with all CIP characteristic diffraction peaks at 14.3°, 20.6°, and 25.2° 2$\theta$ (**Figure S3A**). The reflections characteristic of PCL were observed at 21.3° and 23.7° 2$\theta$. CIP characteristic diffraction peaks were also identified in diffractograms of PMs of PCL and 4% CIP, approximately at 14.3° and 25.2° 2$\theta$. Additionally, 8% CIP content in PM was tested, and it was verified that the identified CIP characteristic diffraction peaks were even more pronounced (data not shown). This finding confirmed that it is possible to detect the presence of crystalline CIP in mixtures with PCL using XRD.

The diffraction patterns of ES PCL-CIP fibers showed reflections specific only to PCL, but interestingly, no clearly distinguished CIP characteristic reflections and only two small diffraction peaks were present at approximately 6.8 and 8.7° 2$\theta$. This indicates that most likely most of the CIP crystal form changed during ES into an amorphous form, which is often reported that the ES process can change the solid state form, and often the amorphous drug is obtained. It has been shown previously that CIP transforms from crystalline to an amorphous form during ES where fast removal of the solvent takes place and molecules do not have time to crystallize [8,9]. However, these additional reflections detected might indicate the presence of some CIP-acetate crystals that have a specific reflection in these ranges previously proposed by Zupančič *et al.* and Uhljar *et al* [2,9] (**Figure S3A**). The latter is explained by the fact that the ES solution used to prepare PCL-CIP fibers was prepared in an AA/FA mixture, and therefore, the acetate and formate salts of CIP may be formed. However, no crystals were visually observed on fibers in SEM images (s.f. **Figures S1** and **S2**), unlike what Zupančič *et al.* reported [2].

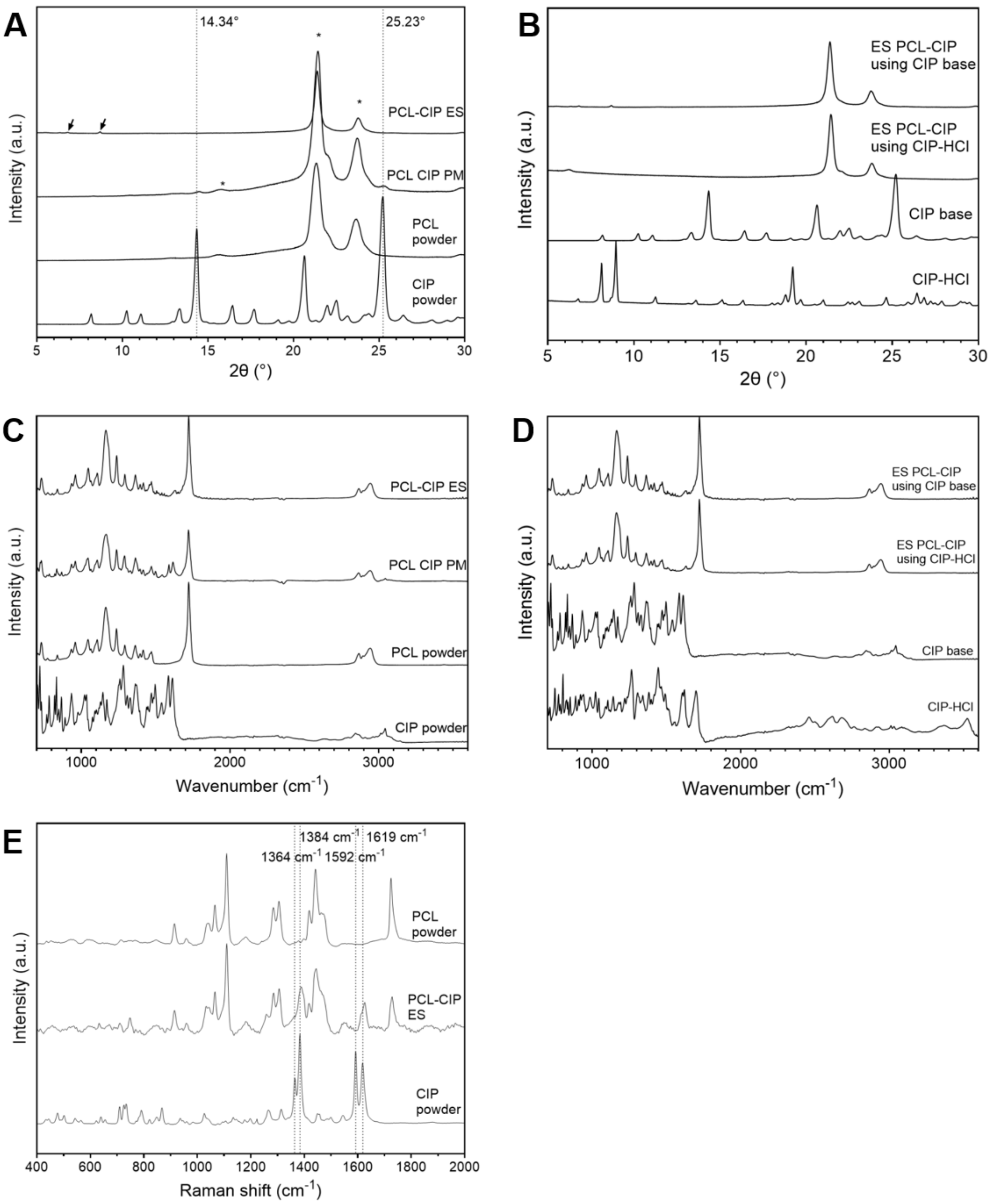


**Figure S3.** Solid state characterization of ES PCL-CIP fibrous dressings. A. XRD diffractograms of ES PCL-CIP fibrous dressing and physical mixture (PM; 4% CIP) and their raw materials. B. XRD diffractograms CIP-HCl and CIP base and ES PCL-CIP fibers prepared using those. C. FT-IR spectra of ES PCL-CIP fibrous dressing and PM and their raw materials. D. FT-IR spectra of CIP-HCl and CIP base and ES PCL-CIP fibers prepared using those.

E. Raman microspectroscopy spectra of ES PCL-CIP fibrous dressing and PM and their raw materials. (N=3). Key: CIP – ciprofloxacin, ES – electrospun fibers, PCL – polycaprolactone, PM – physical mixture. PCL-characteristic diffraction peaks are marked with asterisks (*); CIP-characteristic peaks are marked with dotted lines. Additional small peaks in ES PCL-CIP diffractogram are marked with arrows.

*FT-IR spectroscopy.* The FT-IR spectrum of CIP powder shows characteristic peaks at 1612 $cm^{-1}$ and 1585 $cm^{-1}$ (**Figure S3C**), known to be due to the molecular vibrations related to C=O symmetric vibrations of carboxylate ion and C=O symmetric and asymmetric vibrations of carboxylate ions, respectively [10,11]. In the spectra of PCL and CIP PM and PCL-CIP fibers, the characteristic peaks of PCL dominated. CIP characteristic peaks that did not overlap with PCL ones, were easily distinguished and visible, especially in the range from ~1500 $cm^{-1}$ to ~1700 $cm^{-1}$. The characteristic CIP peaks were more detectable in the spectra of PCL and CIP PMs compared to ES PCL-CIP fiber ones. The CIP characteristic peak at 1375 $cm^{-1}$ was not seen in the spectrum of PCL CIP PM because of the overlap with the PCL spectrum. Still, the peak related to the C=O symmetric and asymmetric vibrations of carboxylate ions was seen at approximately 1589 $cm^{-1}$. However, both of those CIP characteristic peaks were missing in the ES PCL-CIP spectrum, as also shown previously [2,10,11].

Several CIP peaks in the spectrum of ES PCL-CIP were shifted compared to the spectrum of PCL and CIP PM. There was a peak shift observed from 1616 to 1628 $cm^{-1}$. This CIP specific band of C=O symmetric vibrations of carboxylate ion was identified at 1628 $cm^{-1}$ in the spectrum of ES PCL-CIP fibers. The latter has been shown to occur also previously [12]. This could indicate that CIP in its raw material was in zwitterionic or ionic form, whereas CIP was unionized during the ES process. The peak approximately at 1700 $cm^{-1}$, which indicates C=O vibrations of the carboxylate group, was not detectable due to the overlapping with the PCL peak [8].

Furthermore, the band at 3200 $cm^{-1}$ corresponding to O-H stretching is seen for PM, but not for ES fiber spectrum confirming intermolecular interactions between CIP and the carrier polymer in ES fibers. According to the FT-IR spectral and XRD analysis, it was confirmed that despite the different CIP raw materials (base *vs* HCl salt) used for the ES of fibrous drug loaded dressings, CIP solid state form was the same within the fibers (**Figure S3B** and **S3D**).

*Raman microspectroscopy.* RSM measurements proved the findings from FT-IR measurements (**Figure S3E**). The CIP characteristic peaks were identified in the Raman spectra of PCL-CIP ES fibers with small shifts. The peaks at 1592 $cm^{-1}$ and 1619 $cm^{-1}$ in the CIP spectrum, indicating C=O stretching vibrations and C=C stretching vibrations, respectively, were merged into one peak at 1626 $cm^{-1}$ in the ES PCL-CIP spectrum. This indicates that some changes in molecular bonding took place during ES. Another intensive peak from stretching vibrations of the quinolone ring system at 1384 $cm^{-1}$ in the CIP spectrum [12] shifted to 1389 cm-1 in the ES PCL-CIP spectrum. There were no peaks of C=O vibrations of carboxylate ion approximately at 1700 $cm^{-1}$, indicating again that the CIP powder was in zwitterionic form. The solid-state characterization revealed that the PCL-CIP fibrous dressings ES using CIP base in AA:FA solution exhibit similar solid-state properties compared to the fibers ES using CIP-HCL [2]. It was seen that despite the initial CIP solid state form selected for the fiber preparation or additional γ-sterilization of the fibrous dressings, the same solid-state form of CIP was present in the ES fibers.

### 1.2.5 *In vitro* drug release from ES fibrous wound dressings

The drug release from PCL-CIP fibrous dressing showed burst release of CIP from sterilized fibers in PBS at 37 °C (**Figure S4**). Hence, the use of CIP-base instead of CIP-HCl or γ-sterilization did not modify the release of CIP since similar results have been shown previously [2]. Within the first 5 min, approximately 75 % of CIP was released, and by the 1 h time point, all of the CIP was released from the PCL fibrous dressing.

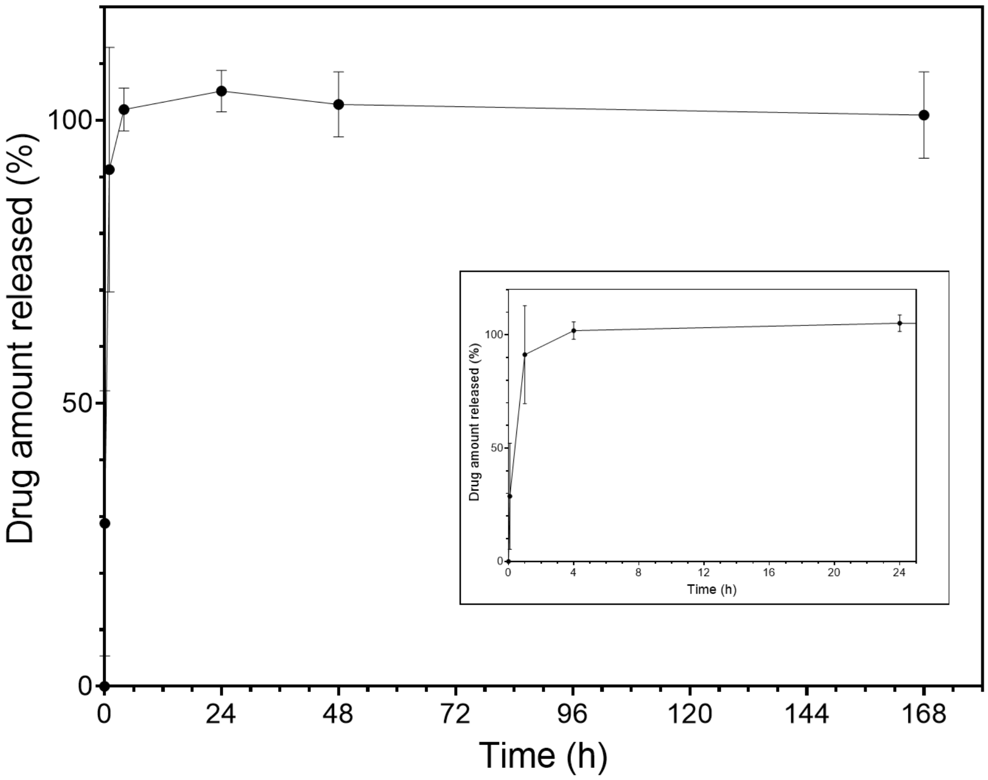


**Figure S4.** Ciprofloxacin (CIP) release from γ-sterilized polycaprolactone (PCL)-CIP fibrous dressing in PBS at 37 °C (N=3). Figure enlargement reveals the burst release and the CIP release up to 24 h of testing.

## 2. Single-species bacterial biofilm models

Single-species bacterial biofilm models as controls were grown for each individual bacterial species (*S. aureus, P. aeruginosa*, and *E. coli*) for 24 h and 48 h, and confocal microscopy images were collected (**Figure S5;** data shown for 24 h samples).

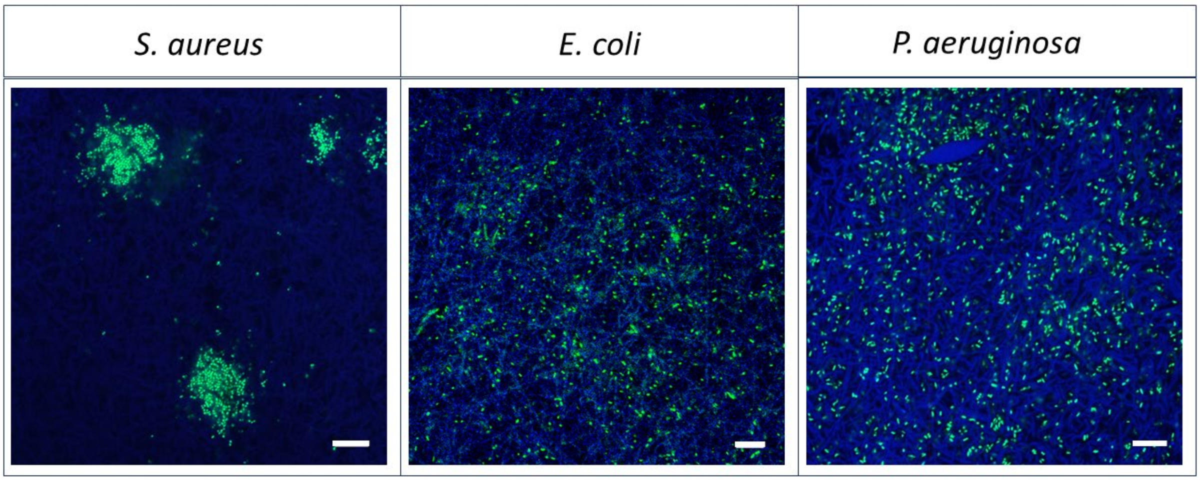


**Figure S5.** Confocal fluorescence microscopy (CFM) images of single-species *in vitro* biofilms of A. *S. aureus,* B. *E. coli* and C. *P. aeruginosa* on Gel-Gluc after 24 h of incubation. Stained with Syto-9™ (green), Gel-Gluc autofluorescence (blue). CFM images scale bar 10 µm.

## 3. Biofilm model validation using drug-loaded ES fibrous wound dressings

All models were validated using previously reported ES antibacterial wound dressings. The mean changes in log CFU compared to the same time point of untreated biofilm development were calculated to determine the log reduction of each sample. All the results are presented as a total number of bacteria on Gel-Gluc and ES fibrous dressing, where applicable. The ratio of bacteria located on one or another substrate was also checked, and it was seen that preferentially biofilm bacteria attached to the Gel-Gluc compared to ES fibrous dressings (**Figure S6**).

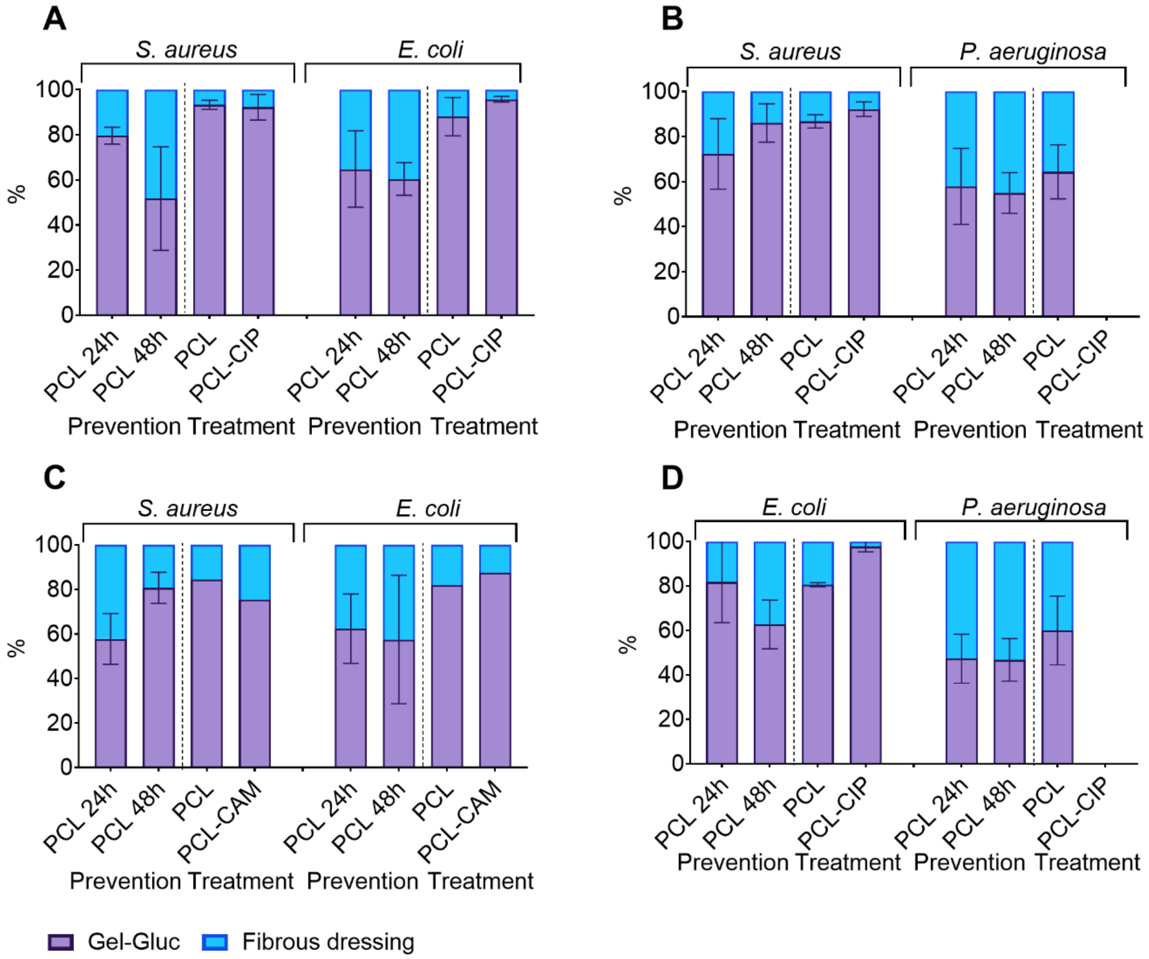


**Figure S6.** Percentage of bacteria measured after 24 h and 48 h of prevention from gelatin-glucose matrix (Gel-Gluc) and pristine electrospun (ES) polycaprolactone (PCL) fibrous dressings, and after 24 h treatment from Gel-Gluc, ES pristine, and antibiotic-containing PCL fibrous dressings. A. *S. aureus* and *E. coli* validation using PCL-CIP formulation. B. *S. aureus* and *P. aeruginosa* validation using PCL-CIP formulation. C. *S. aureus* and *E. coli* validation using PCL-CAM formulation. D. *E. coli* and *P. aeruginosa* validation using PCL-CIP formulation. Key: PCL 24 h and PCL 48 h – PCL treated samples' results of prevention assay after 24 h and 48 h, respectively; PCL, PCL-CIP, PCL-CAM, PCL, respectively, treated samples' results after 24 h of treatment assay. Data are presented as mean and SD.

More bacteria were able to colonize the ES fibrous wound dressings during biofilm prevention testing compared to the biofilm treatment testing. Only *P. aeruginosa* did not show any major differences and was able to colonize both fibrous matrices and fibrous dressings (Gel-Gluc *vs* ES fibrous wound dressings) similarly.

# 3. References


(1) Preem, L.; Mahmoudzadeh, M.; Putrinš, M.; Meos, A.; Laidmäe, I.; Romann, T.; Aruväli, J.; Härmas, R.; Koivuniemi, A.; Bunker, A.; Tenson, T.; Kogermann, K. Interactions between Chloramphenicol, Carrier Polymers, and Bacteria-Implications for Designing Electrospun Drug Delivery Systems Countering Wound Infection. *Mol. Pharm.* **2017**, *14* (12), 4417–4430. https://doi.org/10.1021/acs.molpharmaceut.7b00524.

(2) Zupančič, Š.; Preem, L.; Kristl, J.; Putrinš, M.; Tenson, T.; Kocbek, P.; Kogermann, K. Impact of PCL Nanofiber Mat Structural Properties on Hydrophilic Drug Release and Antibacterial Activity on Periodontal Pathogens. *Eur. J. Pharm. Sci.* **2018**, *122*, 347–358. https://doi.org/10.1016/j.ejps.2018.07.024.

(3) Lorenz, K.; Preem, L.; Sagor, K.; Putrinš, M.; Tenson, T.; Kogermann, K. Development of In Vitro and Ex Vivo Biofilm Models for the Assessment of Antibacterial Fibrous Electrospun Wound Dressings. *Mol. Pharm.* **2023**, *20* (2), 1230–1246. https://doi.org/10.1021/acs.molpharmaceut.2c00902.

(4) Siimon, K.; Reemann, P.; Põder, A.; Pook, M.; Kangur, T.; Kingo, K.; Jaks, V.; Mäeorg, U.; Järvekülg, M. Effect of Glucose Content on Thermally Cross-Linked Fibrous Gelatin Scaffolds for Tissue Engineering. *Mater. Sci. Eng. C* **2014**, *42*, 538–545. https://doi.org/10.1016/j.msec.2014.05.075.

(5) Siimon, K.; Siimon, H.; Järvekülg, M. Mechanical Characterization of Electrospun Gelatin Scaffolds Cross-Linked by Glucose. *J. Mater. Sci. Mater. Med.* **2015**, *26* (1), 37. https://doi.org/10.1007/s10856-014-5375-1.

(6) Palo, M.; Kogermann, K.; Laidmäe, I.; Meos, A.; Preis, M.; Heinämäki, J.; Sandler, N. Development of Oromucosal Dosage Forms by Combining Electrospinning and Inkjet Printing. *Mol. Pharm.* **2017**, *14* (3), 808–820. https://doi.org/10.1021/acs.molpharmaceut.6b01054.

(7) Preem, L.; Vaarmets, E.; Meos, A.; Jõgi, I.; Putrinš, M.; Tenson, T.; Kogermann, K. Effects and Efficacy of Different Sterilization and Disinfection Methods on Electrospun Drug Delivery Systems. *Int. J. Pharm.* **2019**, *567*, 118450. https://doi.org/10.1016/j.ijpharm.2019.118450.

(8) Uhljar, L. É.; Kan, S. Y.; Radacsi, N.; Koutsos, V.; Szabó-Révész, P.; Ambrus, R. In Vitro Drug Release, Permeability, and Structural Test of Ciprofloxacin-Loaded Nanofibers. *Pharmaceutics* **2021**, *13* (4), 556.

(9) Uhljar, L. É.; Alshweiat, A.; Katona, G.; Chung, M.; Radacsi, N.; Kókai, D.; Burián, K.; Ambrus, R. Comparison of Nozzle-Based and Nozzle-Free Electrospinning for Preparation of Fast-Dissolving Nanofibers Loaded with Ciprofloxacin. *Pharmaceutics* **2022**, *14* (8), 1559. https://doi.org/10.3390/pharmaceutics14081559.

(10) Li, H.; Zhang, Z.; Godakanda, V. U.; Chiu, Y.-J.; Angkawinitwong, U.; Patel, K.; Stapleton, P. G.; de Silva, R. M.; de Silva, K. M. N.; Zhu, L.-M.; Williams, G. R. The Effect of Collection Substrate on Electrospun Ciprofloxacin-Loaded Poly(Vinylpyrrolidone) and Ethyl Cellulose Nanofibers as Potential Wound Dressing Materials. *Mater. Sci. Eng. C* **2019**, *104*, 109917. https://doi.org/10.1016/j.msec.2019.109917.

(11) Mesallati, H.; Tajber, L. Polymer/Amorphous Salt Solid Dispersions of Ciprofloxacin. *Pharm. Res.* **2017**, *34* (11), 2425–2439. https://doi.org/10.1007/s11095-017-2250-z.

(12) Neugebauer, U.; Szeghalmi, A.; Schmitt, M.; Kiefer, W.; Popp, J.; Holzgrabe, U. Vibrational Spectroscopic Characterization of Fluoroquinolones. *Spectrochim. Acta. A. Mol. Biomol. Spectrosc.* **2005**, *61* (7), 1505–1517. https://doi.org/10.1016/j.saa.2004.11.014.
